# Integrated computational analysis reveals HOX genes cluster as oncogenic drivers in head and neck squamous cell carcinoma

**DOI:** 10.1038/s41598-022-11590-1

**Published:** 2022-05-13

**Authors:** U Sangeetha Shenoy, Richard Morgan, Keith Hunter, Shama Prasada Kabekkodu, Raghu Radhakrishnan

**Affiliations:** 1 Department of Cell and Molecular Biology, Manipal School of Life Sciences, Manipal Academy of Higher Education, Manipal, 576104 Karnataka India; 2 School of Biomedical Sciences, University of West London, London, W5 5RF UK; 3 Academic Unit of Oral and Maxillofacial Medicine and Pathology, School of Clinical Dentistry, University of Sheffield, Sheffield, S10 2TA UK; 4 Department of Oral Pathology, Manipal College of Dental Sciences, Manipal Academy of Higher Education, Manipal, 576104 India

**Keywords:** Cancer, Computational biology and bioinformatics, Genetics, Molecular biology, Biomarkers, Oncology

## Abstract

Alterations in homeobox (HOX) gene expression are involved in the progression of several cancer types including head and neck squamous cell carcinoma (HNSCC). However, regulation of the entire HOX cluster in the pathophysiology of HNSCC is still elusive. By using different comprehensive databases, we have identified the significance of differentially expressed *HOX* genes (DEHGs) in stage stratification and HPV status in the cancer genome atlas (TCGA)-HNSCC datasets. The genetic and epigenetic alterations, druggable genes, their associated functional pathways and their possible association with cancer hallmarks were identified. We have performed extensive analysis to identify the target genes of DEHGs driving HNSCC. The differentially expressed HOX cluster-embedded microRNAs (DEHMs) in HNSCC and their association with HOX-target genes were evaluated to construct a regulatory network of the HOX cluster in HNSCC. Our analysis identified sixteen DEHGs in HNSCC and determined their importance in stage stratification and HPV infection. We found a total of 55 HNSCC driver genes that were identified as targets of DEHGs. The involvement of DEHGs and their targets in cancer-associated signaling mechanisms have confirmed their role in pathophysiology. Further, we found that their oncogenic nature could be targeted by using the novel and approved anti-neoplastic drugs in HNSCC. Construction of the regulatory network depicted the interaction between DEHGs, DEHMs and their targets genes in HNSCC. Hence, aberrantly expressed HOX cluster genes function in a coordinated manner to drive HNSCC. It could provide a broad perspective to carry out the experimental investigation, to understand the underlying oncogenic mechanism and allow the discovery of new clinical biomarkers for HNSCC.

## Introduction

Head and neck squamous cell carcinoma (HNSCC) is a group of cancers originating from the oral cavity mucosa, larynx, and pharynx^1^. It is the sixth most common cancer globally, with 890,000 new cases and 450,000 deaths recorded in 2018^2^. Despite having several advances in the treatment of HNSCC, mortality remains high due to the absence of a reliable biomarker that would allow early diagnosis. HNSCC is remarkably heterogeneous, and most of the cases are caused by the use of alcohol and smokeless tobacco, with some association with human papillomavirus (HPV) infection^3^. All these factors individually or in combination contribute to an unfavorable clinical outcome and poor prognosis in HNSCC. Hence, the clinical stage of the tumor and the HPV status, however, are regarded as the critical determinants of HNSCC prognosis^4^.

Genetic, epigenetic factors and post-translational modifications of key regulatory genes play a crucial role in tumor progression^5–8^. Hence, the discovery of reliable, robust biomarkers with translational relevance may improve the treatment outcomes in HNSCC. Homeobox (*HOX*) genes are unique in that several of the aberrantly expressed protein-coding genes and noncoding RNAs (ncRNAs) may serve as biomarkers for early cancer detection^9,10^. They play a dynamic role in mammalian development by determining the identity of specific segmental regions of the body^11^. A total of 39 *HOX* genes located on four different clusters form a complex regulatory network, and aberrant *HOX* gene expression has been implicated in abnormal development and malignancy^12–15^.

The biological significance of the genetic and epigenetic changes in the HOX cluster genes in cancer has been extensively reviewed^16^. According to the studies, *HOX* genes are widely regulated by epigenetic factors such as promoter DNA methylation and ncRNAs^14,17–19^. The majority of *HOX* genes harbors unique ncRNAs within their 3' untranslated region (UTR)^19^. Characterization of HOX loci revealed 231 HOX cluster-embedded ncRNAs that spanned the known transcribed region by more than 30 kilobases (kb), with broad implications for both development and disease^20^. Several conserved noncoding sequences act as cis-regulatory elements that regulate the expression of orthologous and paralogous *HOX* genes^21^. The coordinated regulatory interaction between members of each HOX cluster and their downstream biological targets is necessary for the proper functioning of organs at the tissue and cellular levels. Hence, aberrant expression of *HOX* genes and associated ncRNAs may contribute to abnormal proliferation, migration, invasion, metastasis, and epithelial-mesenchymal transition (EMT)^19,22,23^. Studies to decode the regulatory mechanism of the entire HOX cluster may be beneficial for a thorough understanding of its oncogenic potential and for identifying their potential as driver genes in HNSCC. It would also help to determine, to what extent the oncogenic mechanism might be driven not only by individual *HOX* genes but also by the deregulation of multiple genes^24^.

In this paper, we have identified the differentially expressed *HOX* genes (DEHGs) using the cancer genome atlas (TCGA) HNSCC datasets. The extensive bioinformatics analysis determined their genetic and epigenetic alterations, diverse downstream targets, biological functions, and clinicopathological significance. We constructed the HOX cluster regulatory network by identifying the differentially expressed HOX cluster-embedded microRNAs (DEHMs) and predicting their potential targets in HNSCC. Experimental validation of the regulatory interactome of HOX clusters may enhance our understanding of underlying molecular pathology. Moreover, the findings of our study may allow the discovery of potential biomarkers for prognosis and guided therapy in HNSCC.

## Results

### Differentially expressed HOX genes (DEHGs) in head and neck squamous cell carcinoma

**Figure 1:**
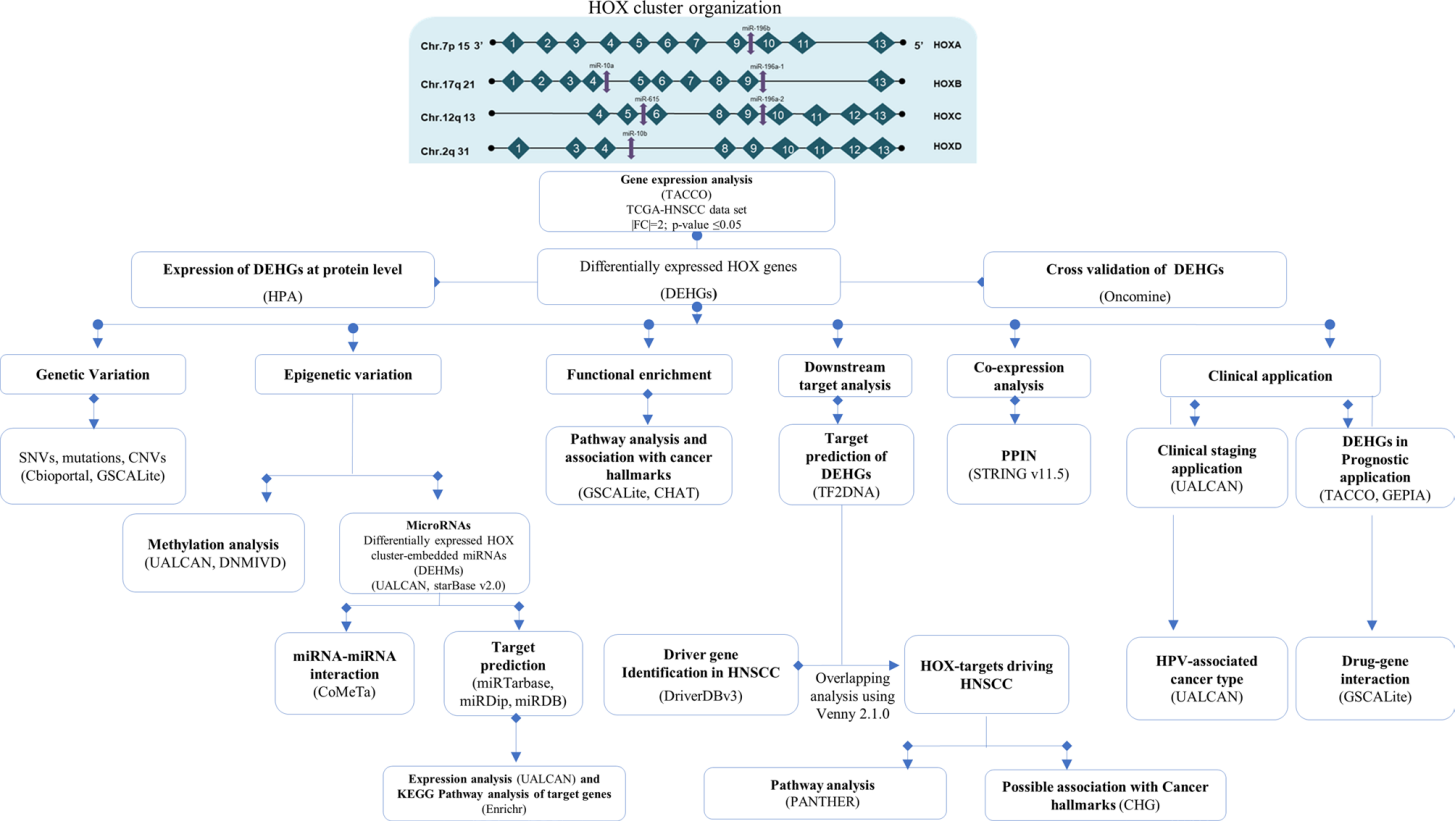
The workflow of in silico analysis of the *HOX* genes and HOX-embedded miRNA network in Head and Neck Squamous Cell Carcinoma (HNSCC).

The detailed workflow involved in retrieving and analyzing differentially expressed genes (DEGs) and their corresponding results is depicted in Fig. 1 and Table 1 respectively. A total of 1322 DEGs from the TCGA-HNSCC dataset was retrieved from the Transcriptome Alterations in Cancer Omnibus^25^ (TACCO, http://tacco.life.nctu.edu.tw/) database, of which 460 genes were upregulated and 862 were downregulated with log2 fold change of  >  + 2 and <  − 2 between tumor (n = 520) and normal (n = 44) tissue samples (*p*-value ≤ 0.05). Among these, 16 HOX cluster genes (Supplementary Table  S1) were found to be upregulated in the TCGA-HNSCC dataset (*p*-value of ≤ 0.05).

**Table 1:** The differentially expressed *HOX* genes in association with head and neck squamous cell carcinoma.

DEHGs	Genes
Upregulated	*HOXA9, HOXA10, HOXA11, HOXB7, HOXB9, HOXB13, HOXC4, HOXC5, HOXC6, HOXC8, HOXC9, HOXC10, HOXD1, HOXD10, HOXD11, HOXD13*
Differentially methylated	*HOXA9, HOXA10, HOXA11, HOXB7, HOXB13, HOXC8, HOXC9, HOXD10, HOXD13*
Hypermethylated genes	*HOXD10*
Inverse correlation—Gene expression and methylation	*HOXA9, HOXA10, HOXB7, HOXB13, HOXC9*
Positive correlation—Gene expression and methylation	*HOXD10*
SNVs	Missense mutation: *HOXA11, HOXB7, HOXB13, HOXC4, HOXC6, HOXC8, HOXC9, HOXC10, HOXD10, HOXD13* Non-sense mutation: *HOXB7* In-frame deletion: *HOXD13*
CNVs	*HOXA9, HOXA10, HOXA11, HOXB7, HOXB9, HOXB13, HOXC4, HOXC5, HOXC6, HOXC8, HOXC9, HOXC10, HOXD1, HOXD10, HOXD11, HOXD13*
Associated with HPV infection	DE between HPV- positive and HPV-negative tumors: *HOXB13, HOXC5, HOXC6, HOXC9* and *HOXD11*
Cancer staging	Stage 1 versus stage 4: *HOXA10*, *HOXB9,* and *HOXC8* Stage 2 versus stage 4: *HOXC4* All the stages: *HOXD1*
Survival status	OS- *HOXC5, HOXC6, HOXB9* DFS- *HOXC8*

All the genes mentioned in this table are statistically significant in the corresponding analysis performed (*p* ≤ 0.05). Supplementary Tables  S1–S6 shows the fold change values, sample size, and statistical significance of all the analysis performed.

### Genetic variations associated DEHGs

**Figure 2:**
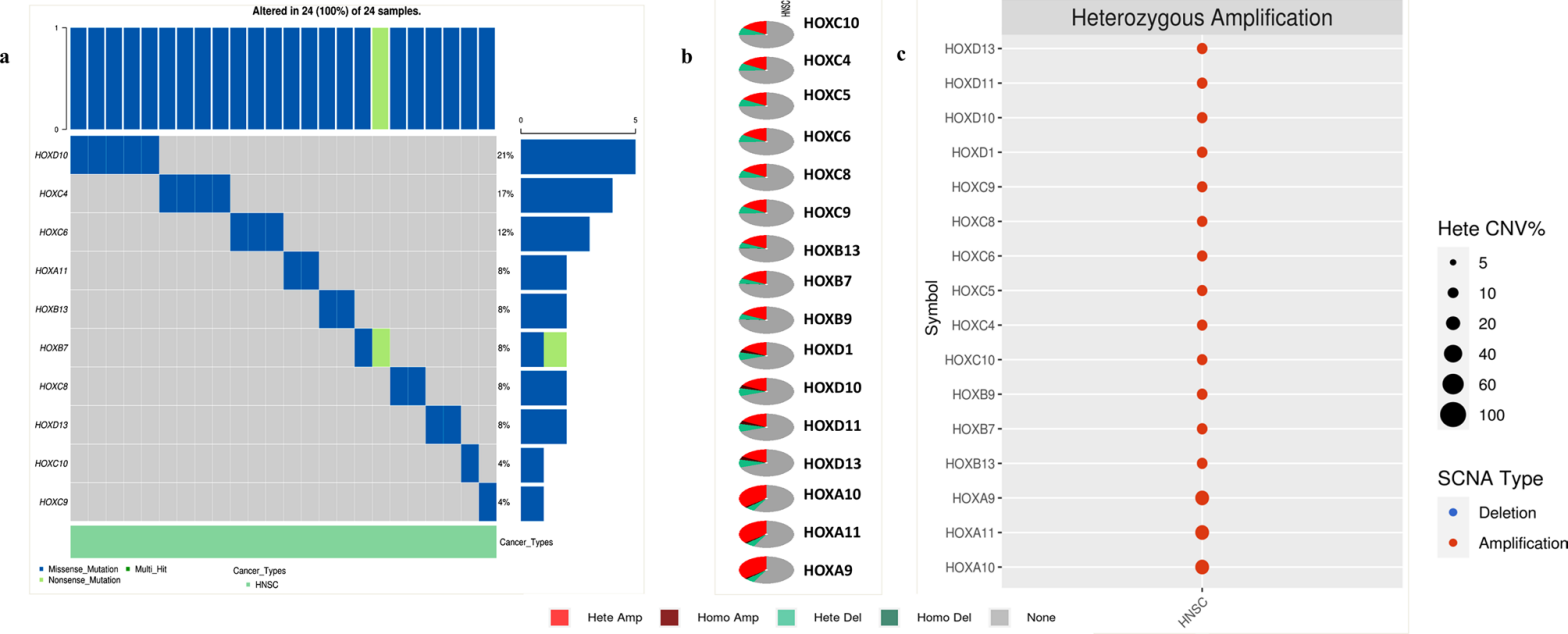
Differentially expressed *HOX* genes (DEHGs) and their genetic variation in HNSCC: (**a**) Single Nucleotide Variants (SNV): Oncoplot showing the top ten mutated genes in the HNSCC data set. The type of SNV is color-coded as shown in the figure. Bar plots on the side and top of the figure, show the number of variants in each sample and gene respectively. (**b**) Copy Number Variation (CNV): Pie chart represents the global profile that shows the proportion of heterozygous or homozygous CNV of each gene in HNSCC. c) CNV profile shows the percentage of heterozygous amplification, about each gene with > 5% CNV in HNSCC. Red color bubble intensity represents the positive correlation between higher gene expression levels and the high frequency of CNVs. The size of the point positively correlates with statistical significance.

We have determined the genetic variations in DEHGs using Gene Set Cancer Analysis^26^ (GSCALite, http://bioinfo.life.hust.edu.cn/web/GSCALite/). Of the 16 DEHGs identified, *HOXA11, HOXB7, HOXB13, HOXC4, HOXC6, HOXC8, HOXC9, HOXC10, HOXD10, HOXD13* showed missense mutations, *HOXB7* had a nonsense mutation, and *HOXD13* had an in-frame deletion in the HNSCC tissue samples (n = 24) (Fig. 2a, b). Similar trends were observed in the tissue samples (n = 530; Supplementary Table S2) retrieved from TCGA-firehose legacy HNSCC dataset accessed using CBioPortal^27^ (https://www.cbioportal.org/). Moreover, this observation was only evident in a few samples analyzed, suggesting the involvement of (epi) genetic players other than SNVs and mutations. Notably, all the DEHGs had copy-number variations (CNVs) in HNSCC when analyzed using the GSCALite tool. Heterozygous amplification ranged from 20 to 40%, with the highest amplification was noted in *HOXA9*, *HOXA10*, and *HOXA11* (Fig. 2c).

### Alteration in DNA methylation associated with DEHGs

Changes in DNA methylation associated with DEHGs were assessed using the UALCAN^28^ (http://ualcan.path.uab.edu/) and DNA Methylation Interactive Visualization Database^29^ (DNMIVD; http://119.3.41.228/dnmivd/index/). Out of the 16 DEHGs, nine genes showed a marked difference in the methylation profile between the normal (n = 50) and tumor samples (n = 528) in the TCGA-HNSCC dataset having a *p*-value of ≤ 0.05 (Supplementary Table  S3). Of these genes, HOXD10 showed significant hypermethylation. In comparison, five genes showed an inverse correlation between gene expression and promoter DNA methylation (Supplementary Fig. S1).

### Cross-validation of DEHGs in independent datasets and the expression of DEHGs at the protein level

The findings of our analysis were cross-validated using the Oncomine tool consisting of 264 independent datasets across 35 cancer types^30^ (https://www.oncomine.org/resource/login.html). Oncomine analysis identified seven genes namely *HOXA9*^31,32^, *HOXA10*^31,33,34^, *HOXB7*^32–36^, *HOXC6*^31,33–35,37^, *HOXC9*^31,35^, *HOXC10*^34^ and *HOXD10*^35,36,38^ that were upregulated in more than 2 independent HNSCC datasets (Supplementary Fig. S2).

The immunohistochemical (IHC) data retrieved from the Human Protein Atlas (HPA) database^39^ (HPA: http://www.proteinatlas.org/) was available only for eight DEHGs. Except for *HOXB7*, the expression of *HOXA9, HOXA10, HOXA11, HOXB13, HOXC5, HOXC8,* and *HOXC10* at the protein level was following their mRNA expression level (Supplementary Fig. S3).

### Construction of HOX protein interaction network

**Figure 3:**
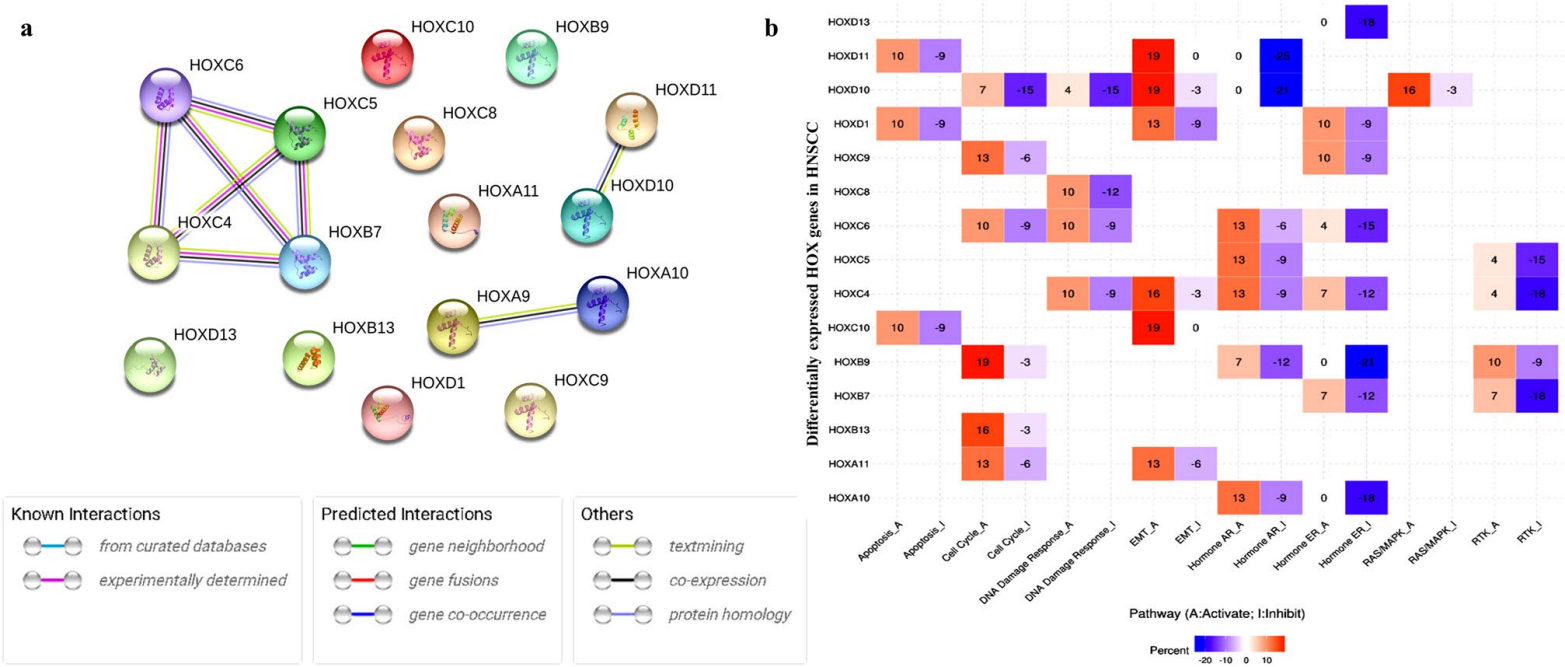
Co-expression analysis: (**a**) Overview of PPI Network of 16 DEHGs, with > 0.7 confidence score constructed using STRING v11.5 database. The network includes 8 edges between 16 nodes that show the co-expression amongst DEHGs. (**b**) Pathway analysis: A heatmap showing DEHGs that activate (A) and inhibit (I) pathways in HNSCC using GSCALite.

The protein–protein interaction network (PPIN) of the candidate genes was constructed using the Search Tool to Retrieve Interacting Genes^40^ (STRING, https://string-db.org) version 11.5. The STRING analysis identified 16 nodes and 8 edges with a PPIN enrichment *p*-value of < 1.0e−16 (Fig. 3a). From the analysis, we found the possible interaction among the HOX proteins, namely, HOXC4, HOXC5, HOXC6, and HOXB7. Since there is experimental evidence that demonstrated the upregulation of *HOXC4, HOXC5, HOXC6,* and *HOXB7* genes and their biological implications in HNSCC clinical samples^41–44^, our study provides the basis to experimentally validate their co-expression and determine their biological significance in HNSCC.

### Association of DEHGs in the oncogenic pathways

Analysis of DEHGs in oncogenic pathways using GSCALite^26^ indicated that *HOXD10, HOXD11, HOXD1, HOXC4, HOXC10,* and *HOXA11* may have a crucial role in the activation of epithelial-mesenchymal transition (EMT) in cancer, represented in the form of heatmap percentage. Abnormal expression of *HOXC10*, *HOXD1* and *HOXD11* has been shown to regulate apoptosis pathways and *HOXC4* in the DNA-damage response. Likewise, *HOXC9, HOXC6, HOXB9, HOXB13,* and *HOXA11* activated the cell cycle. The other DEHGs which were inhibitory included *HOXB7*, *HOXC4,* and *HOXC5* that may be involved in the receptor tyrosine kinase pathway, *HOXD13, HOXC6, HOXC4*, *HOXA10, HOXB7*, and *HOXB9* in the estrogen receptor pathway, and *HOXD10* and *HOXD11* in the androgen receptor pathway (Fig. 3b). Since some of these observations have been experimentally validated in previous studies^45–50^, our analysis confirms the role of DEHGs in oncogenic pathways.

The association of DEHGs with cancer hallmarks was tested using the Cancer Hallmarks Analytics Tool^51^ (CHAT, http://chat.lionproject.net.). We found that ten out of the 16 DEHGs were associated with at least five cancer hallmarks, including invasion and metastasis, immune suppression, cellular energetics, replicative immortality, growth suppressor evasion, genome instability and mutation, angiogenesis, resisting apoptosis, prolonged proliferative signaling, and inflammation (Table. 2).

**Table 2:** Differentially expressed *HOX* genes and their target genes associated with the development of cancer hallmarks.

No	Cancer hall marks	DEHGs	Target genes
1	Activating invasion and metastasis	*HOXA9, HOXA10, HOXA11, HOXB7, HOXB9, HOXB13, HOXC4, HOXC5, HOXC6, HOXC8, HOXC9, HOXC10, HOXD1, HOXD10, HOXD11*	*KRAS, CD226, PIK3R1, MAP2K4, AKT1, ANK2, BRAF, CREBBP, PTPRM*
2	Evading growth suppressors	*HOXA9, HOXA10, HOXA1, HOXB7, HOXB9, HOXB1, HOXC8, HOXC9, HOXC10, HOXD10*	*KRAS, PIK3R1, AKT1, CREBBP*
3	Inducing angiogenesis	*HOXA9, HOXA10, HOXB7, HOXB9, HOXB13, HOXC8, HOXC9, HOXD1, HOXD10*	*IL18, CCR2, IFNE, KRAS, PIK3R1, AKT1, ANK2, BRAF*
4	Sustaining proliferative signaling	*HOXA9, HOXA10, HOXA11, HOXB7, HOXB9, HOXB13, HOXC4, HOXC8, HOXC9, HOXC10, HOXD1, HOXD10*	*IFNE, KRAS, PIK3R1, MAP2K4, AKT1, ANK2, BRAF, CREBBP, PTPRM*
5	Evading immune destruction	–	*CASP8, IL18, CCR2, KRAS, PIK3R1, MAP2K4, AKT1, BRAF*
6	Genome instability and mutation	*HOXA9, HOXA10, HOXA11, HOXB7, HOXB9, HOXB13, HOXC8, HOXC9, HOXC10, HOXD1, HOXD10, HOXD11, HOXD13*	*CASP8*
7	Resisting cell death	*HOXA9, HOXA10, HOXA11, HOXB7, HOXB9, HOXC6, HOXC9, HOXC10, HOXD10*	*CASP8, IL18, CCR2, IFNE, KRAS, MAP2K4, AKT1, BRAF, CREBBP*
8	Tumor-promoting inflammation	*HOXB7, HOXC9*	*CASP8, CCR2, KRAS, CD226, PIK3R1, MAP2K4, AKT1, BRAF, PTPRM*
9	Enabling replicative immortality	*HOXA9, HOXB7,*	*KRAS, PIK3R1, AKT1, CREBBP*
10	Reprogramming energy metabolism	*HOXA11, HOXD13*	*KRAS, PIK3R1, PC, AKT1, BRAF, CREBBP*

### Targets of the DEHGs, HNSCC-driver genes, pathway analysis, and cancer hallmarks

A total of 263 driver genes in HNSCC were downloaded from the DriverDBv3 database^52^ (http://driverdb.tms.cmu.edu.tw/). For each of the 16 DEHGs, their transcription factor binding motifs and downstream targets were retrieved by inputting the HUGO gene nomenclature committee (HGNC) gene symbol into the TF2DNA database^53^ (http://www.fiserlab.org/tf2dna_db/index.html). The downstream targets of DEHGs driving HNSCC are summarized in Supplementary Table S4. Amongst 263 driver genes, we found 55 genes that were targets of DEHGs, out of which caspase-8 (*CASP8)*, interleukin-18 (*IL-18*), and transcription factor dimerization partner 2 (*TFDP2*) were identified as the most common targets of the DEHGs in HNSCC.

HOX-regulated downstream targets were analyzed using the Protein Analysis Through Evolutionary Relationships (PANTHER, http://www.pantherdb.org/) webserver^54,55^. Pathways enrichment analysis of the 55 target genes suggested enrichment of 36 cancer-related pathways including angiogenesis, EGFR receptor signaling, integrin signaling, Ras-signaling, and VEGF signaling pathways (Table 3). In addition, how these targets influence and regulate the acquisition of cancer hallmarks were annotated using the cancer hallmark genes (CHG, http://bio-bigdata.hrbmu.edu.cn/CHG/) database^56^ (Table 2).

**Table 3:** Target genes of the differentially expressed *HOX* genes in HNSCC determining the oncogenic signaling pathways.

No	Targets of DEHGs	Signaling pathway
1	*KRAS, MAP2K4, AKT1, PIK3R1, BRAF*	Angiogenesis (P00005)
2	*CASP8, MAP2K4, AKT1,*	Apoptosis signaling pathway (P00006)
3	*BRAF*	B cell activation (P00010)
4	*PCDHB4*	Cadherin signaling pathway (P00012)
5	*MAP2K4, AKT1, PIK3R1, BRAF*	CCKR signaling map (P06959)
6	*PANK2*	Coenzyme A biosynthesis (P02736)
7	*KRAS, MAP2K4, AKT1, BRAF*	EGF receptor signaling pathway (P00018)
8	*AKT1, PIK3R1,*	Endothelin signaling pathway (P00019)
9	*MAP2K4, AKT1, CASP8*	FAS signaling pathway (P00020)
10	*KRAS, MAP2K4, AKT1*	FGF signaling pathway (P00021)
11	*MAP2K4, AKT1, PIK3R1, CREBBP*	Gonadotropin-releasing hormone receptor pathway (P06664)
12	*CREBBP*	Hedgehog signaling pathway (P00025)
13	*CREBBP*	Heterotrimeric G-protein signaling pathway-Gi alpha and Gs alpha mediated pathway (P00026)
14	*AKT1, PIK3R1, CREBBP*	Hypoxia response via HIF activation (P00030)
15	*KRAS, CCR2, AKT1, BRAF*	Inflammation mediated by chemokine and cytokine signaling pathway (P00031)
16	*MAP2K4*	Insulin/IGF pathway-mitogen activated protein kinase kinase/MAP kinase cascade (P00032)
17	*AKT1, PIK3R1*	Insulin/IGF pathway-protein kinase B signaling cascade (P00033)
18	*KRAS, MAP2K4, PIK3R1, BRAF*	Integrin signaling pathway (P00034)
19	*AKT1, BRAF*	Interleukin signaling pathway (P00036)
20	*SHANK2*	Ionotropic glutamate receptor pathway (P00037)
21	*FBXW7*	Notch signaling pathway (P00045)
22	*MAP2K4*	Oxidative stress response (P00046)
23	*MAP2K4*	p38 MAPK pathway (P05918)
24	*CREBBP, AKT1, PIK3R1*	p53 pathway (P00059)
25	*AKT1*	p53 pathway by glucose deprivation (P04397)
26	*KRAS, AKT1, PIK3R1*	p53 pathway feedback loops 2 (P04398)
27	*KRAS, PIK3R1, BRAF*	PDGF signaling pathway (P00047)
28	*KRAS, AKT1, PIK3R1*	PI3 kinase pathway (P00048)
29	*PC*	Pyruvate metabolism (P02772)
30	*KRAS, MAP2K4, AKT1, BRAF*	Ras pathway (P04393)
31	*BRAF, AKT1, PIK3R1*	T cell activation (P00053)
32	*CREBBP, KRAS*	TGF-beta signaling pathway (P00052)
33	*MAP2K4*	Toll receptor signaling pathway (P00054)
34	*CREBBP*	Transcription regulation by bZIP transcription factor (P00055)
35	*KRAS, PI3KR1, AKT1, BRAF*	VEGF signaling pathway (P00056)
36	*PCDHB4, CREBBP*	Wnt signaling pathway (P00057)

### Association of DEHGs with HPV infection, tumor staging, and prognosis

About 85% of HNSCC samples in the TCGA cohort were HPV-negative^57^. However, a recent study found an association between HNSCC and high-risk HPV infection^58^. Various genetic alterations have been found unique to HPV-positive oral squamous cell carcinoma (OSCC), including distinctive mutational signatures, overall mutational burden, frequent copy number changes, and candidate driver events^59^. The abnormal expression of *HOX* genes has been positively correlated with HPV infection in HPV-associated cancer types such as cervical squamous cell carcinoma (CESC) and HNSCC^48,60,61^. With regards to the HPV-associated HNSCC, *HOXB13, HOXC5, HOXC6, HOXC9, and HOXD11* were differentially expressed between HPV-positive and HPV-negative HNSCC (*p* < 0.05, Supplementary Table S5).

Several studies have identified the changes in the *HOX* gene expression levels between different clinical stages of cancer^15,62^. Our analysis found that DEHGs namely *HOXA10, HOXB9, and HOXC8,* which could distinguish stage 1 from stage 4, and *HOXC4* could distinguish stage 2 from stage 4 (*p* < 0.05, Supplementary Table S6). The *HOXD1* gene was found to be differentially expressed in more than one stage, and its differential expression varied significantly from stage 1 through 4 (*p* < 0.05, Supplementary Table S6).

**Figure 4:**
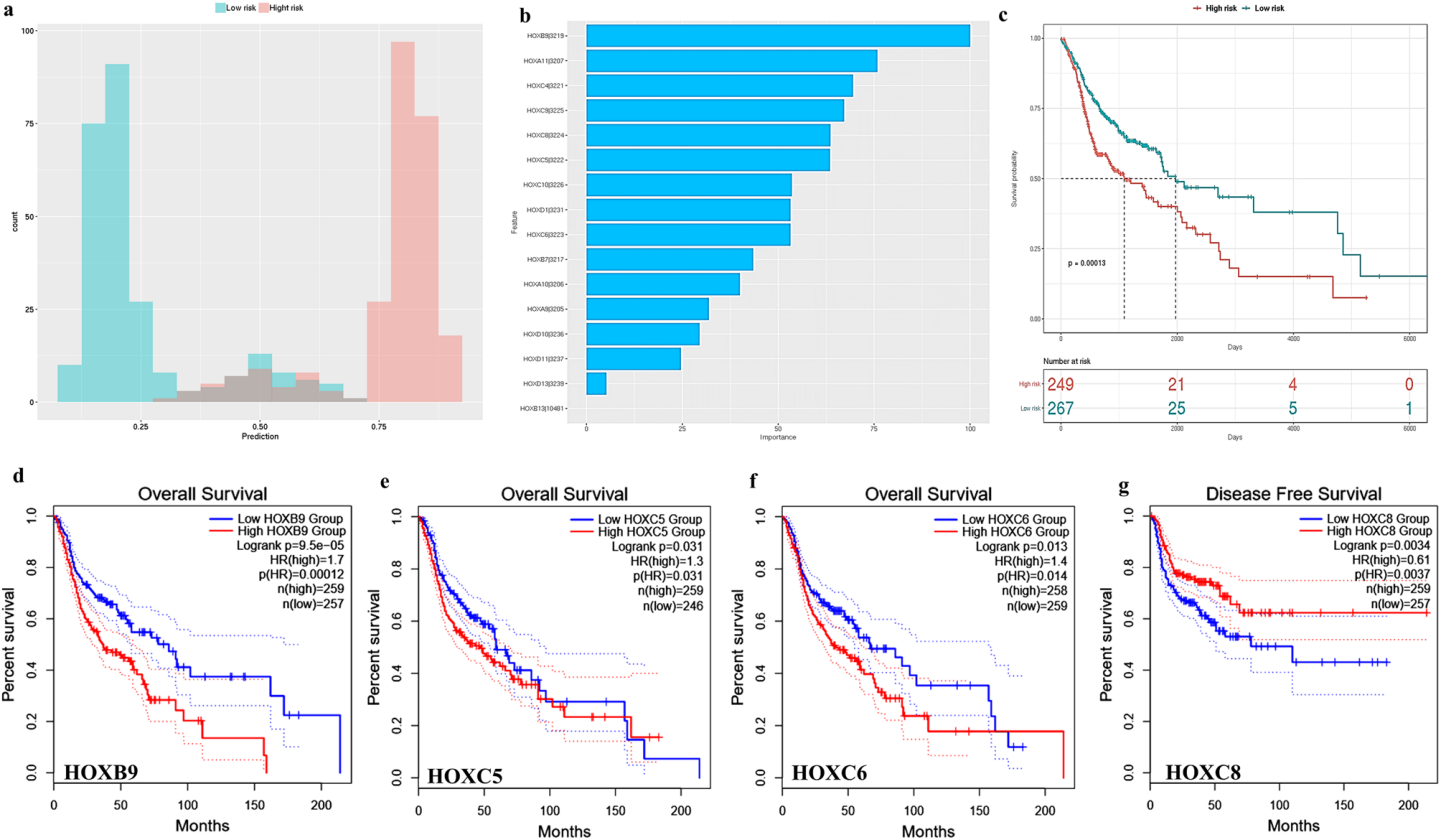
Survival analysis of DEHGs: (**a**) Represents the survival analysis of DEHGs by constructing the prediction model using Random Forest Algorithm: Patients with prediction score larger than 0.5 is considered as high risk, while lower than 0.5 is classified under low risk using log-rank test. (**b**) The contribution of each DEHGs to HNSCC prognosis. (**c**) The Kaplan–Meier survival plot represents the number of patients surviving at each specific time point. (**d-g**) Kaplan–Meier plot of DEHGs associated with overall survival (OS) and disease-free survival (DFS).

TACCO tools with default parameters were used for risk stratification and the determination of the prognostic value of DEHGs in HNSCC. The random-forest model was applied to generate a risk prediction prognostic model for 16 DEHGs, using the TCGA-HNSCC dataset (Fig. 4a). The patients with HNSCC (n = 520) could be categorized into high-risk (n = 249) from low-risk types (n = 267) with a sensitivity of 0.9 and specificity of 0.93, respectively.

In the context of risk stratification, the importance of each of the aberrantly expressed *HOX* genes is shown in Fig. 4b, and survival analysis based on the expression of DEHGs is shown as a Kaplan–Meier (KM) plot (Fig. 4c). Overexpression of *HOXC5, HOXC6,* and *HOXB9* was associated with shorter overall survival (OS) (Fig. 4d–f), while high *HOXC8* expression was significantly associated with longer disease-free survival (DFS) as analyzed by Cox Proportional-Hazards Model using gene expression profiling interactive analysis^63^ (GEPIA2, http://gepia2.cancer-pku.cn/#index) (Fig. 4g). The survival plots describing the OS and DFS of all the DEHGs are presented as Supplementary Fig. S4 and Supplementary Fig. S5.

### Interaction between DEHGs and drug sensitivity

**Figure 5:**
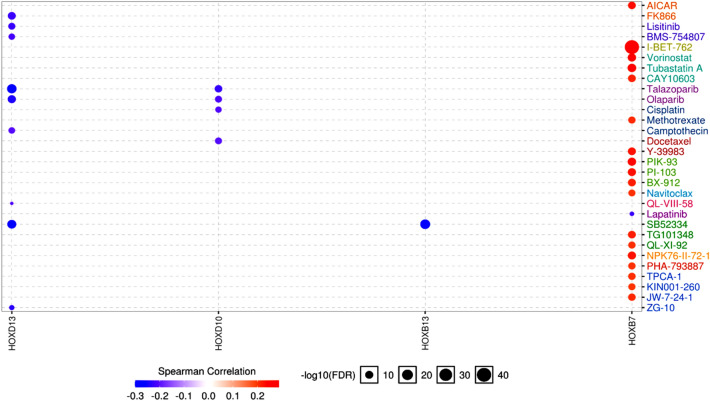
*HOX* genes and drug sensitivity: A bubble plot showing the interaction between the DEHGs and the known and novel therapeutic drugs.

Previously it has been suggested that HOX family of genes play a crucial role in the oncogenic progression of HNSCC and thus could function as a candidate for targeted therapy^64^. Hence, we performed a drug-gene interaction analysis to identify the potential drugs that can be used for targeting the *HOX* gene network. The interaction between DEHGs and 265 approved, anti-neoplastic, and immunotherapeutic agents were analyzed using the genomics of drug sensitivity in cancer^26,65,66^ (GDSC, https://www.cancerrxgene.org/), in GSCALite. The correlation between the expression of each gene in the gene set was measured against the small molecule/drug sensitivity (IC50) using a Spearman correlation analysis. The gene set-drug resistance analysis revealed *HOXB7* to be positively correlated to most known and novel agents, as represented on the bubble plot, which meant that higher expressions of *HOXB7* were associated with drug resistance (Fig. 5). However, a negative correlation was found between *HOXD13, HOXD10,* and *HOXB13* and drug resistance, implying the overexpression of these not only showed sensitivity to strong chemotherapy drugs such as cisplatin and docetaxel but also to the potent oral PARP inhibitors namely talazoparib and olaparib. In addition, *HOXD13* showed sensitivity towards camptothecin, a topoisomerase inhibitor.

### Differentially expressed HOX embedded miRNAs (DEHMs) in HNSCC

**Figure 6:**
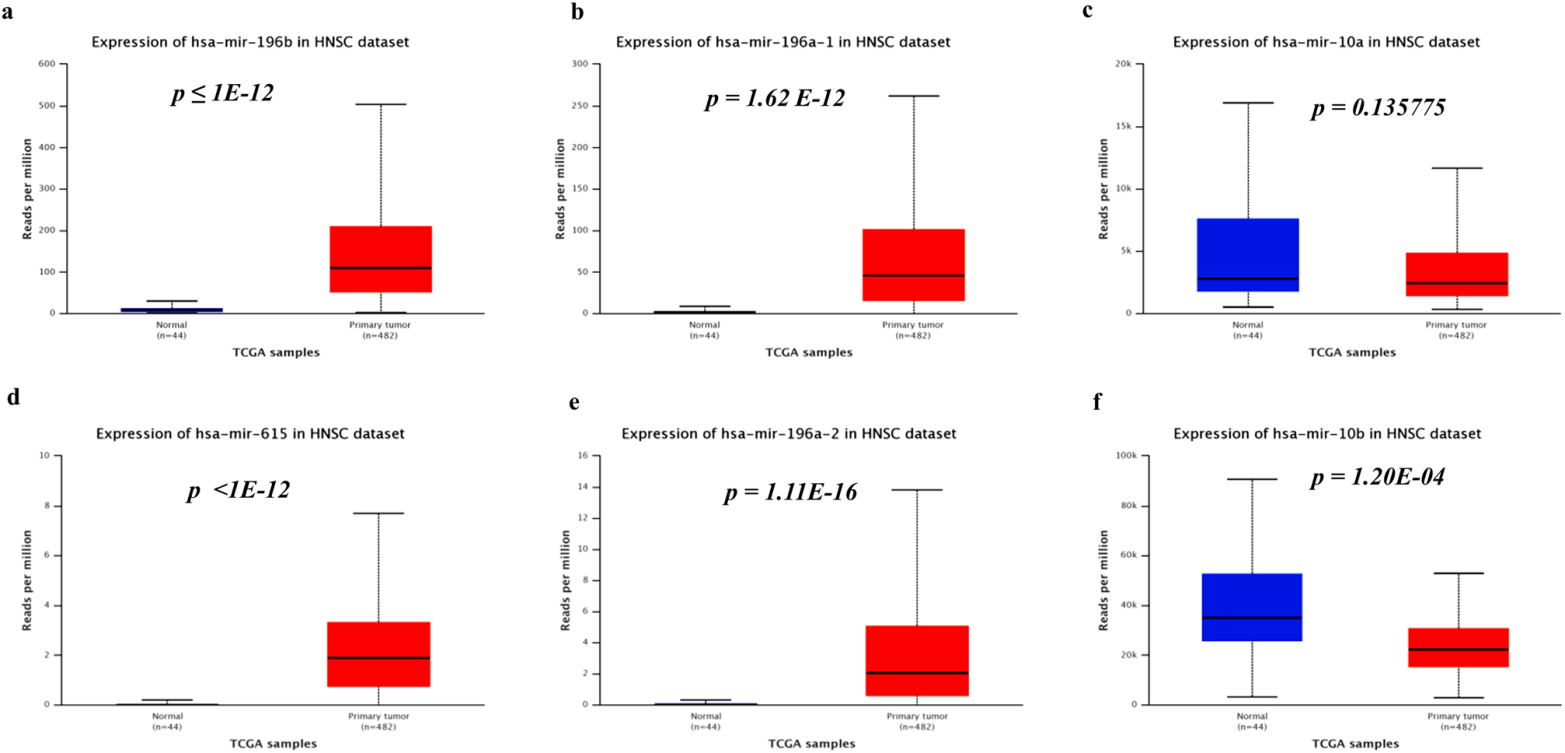
DEHMs in HNSCC: (**a**–**f**) The box plots representing the gene expression of HOX-embedded miRNAs in HNSCC, analyzed using UALCAN.

Aberrantly expressed microRNAs (miRNAs) contribute to the acquisition of cancer hallmarks and serve as a reliable biomarker for early cancer detection^67^. The deregulation of HOX cluster-embedded miRNAs and their corresponding HOX and non-HOX targets lead to cancer progression^68–70^. The present study found that out of six HOX-embedded miRNAs; five were differentially expressed in the TCGA-HNSCC dataset as analyzed using UALCAN (Fig. 6a–f). The miRNAs, miR-196b, miR-196a-1, miR-615, and miR-196a-2, were significantly upregulated (*p* ≤ 0.05) in HNSCC. Whereas miR-10b was downregulated in HNSCC when compared to the normal tissues, there were no significant differences in miR-10a expression.

### DEHMs and their target genes regulating oncogenic pathways

Upon target prediction, we found 108 upregulated target genes and 25 downregulated target genes by analyzing all the five DEHMs in HNSCC (Supplementary Table S7). It is interesting to note that some of the target genes of DEHMs were driver genes of HNSCC (Data from driverDBv3). Further, the Co-expression Meta-analysis of miRNA Targets (CoMeTa) tool^71^ (https://cometa.tigem.it/) identified the co-expression of miR-196a and miR-196b as well as miR-10a and miR-10b. However, the co-expressed miRNAs shared many common target genes.

**Figure 7:**
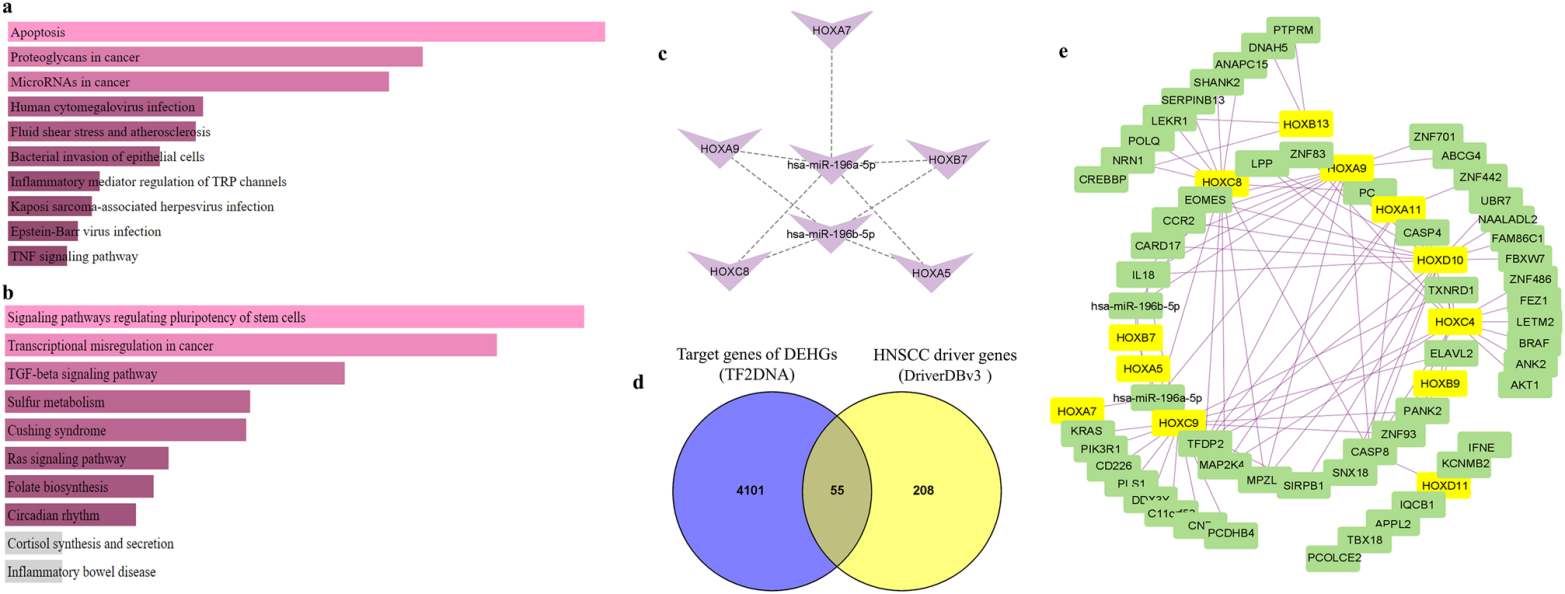
Functional role of DEHMs and their interactive network: (**a**) KEGG pathway analysis of upregulated targets of DEHMs. (**b**) KEGG pathway analysis of downregulated targets of DEHMs. (**c**) Interaction between DEHGs and DEHMs. (**d**) Venn diagram showing the targets of DEHGs driving HNSCC. (**e**) Functional regulatory network of entire HOX cluster genes, their targets, and DEHMs constructed using Cytoscape. The network includes nodes representing DEHGs (yellow squares) and interaction targets (green squares) derived from publicly available databases.

To better understand the functional pathways regulated by target genes of DEHMs, we have performed pathway enrichment analysis using The Kyoto Encyclopedia of Genes and Genomes 2021^73^ (KEGG, https://www.kegg.jp/) pathway accessed using the Enrichr tool^72^ (https://maayanlab.cloud/Enrichr/). Pathway analysis of targets of DEHMs showed that the upregulated target genes were associated with pathways such as miRNAs in cancer, proteoglycans in cancer, apoptosis pathway, Rap1 signaling, TNF signaling, human cytomegalovirus infection, and fluid shear stress (Fig. 7a). The downregulated target genes were enriched in signaling pathways related to regulating pluripotency of stem cells, transcriptional misregulation in cancer, TGF-β signaling, sulfur metabolism, folate biosynthesis, Ras signaling, and Cushing syndrome (Fig. 7b).

The oncogenic potential of DEHMs is not limited to regulating non-HOX targets but is also actively involved in the modulation of *HOX* gene expression in HNSCC. The miR-196 genes were transcribed in three different paralogous locations in the HOX cluster and played a significant role in the temporal activation of *HOXA10, HOXD10*, and *HOXD11*^74^. Notably, co-expression was found between miR-196b and *HOXA7, HOXA10*, and *HOXA11* genes; between miR-196a-2 and *HOXD8* (inverse-correlation); and between miR-196-a1 and *HOXB7* and *HOXB8* genes^75^. Paralogous genes of miR-10 have been found close to HOX4 paralogues; *HOXA1, HOXA3*, and *HOXD10* are targets of the miR-10 family^76^. Notably, miR-615 located in the intron region of the *HOXC5* regulates its expression in a forward-feedback loop resulting in the negative regulation of hTERT mRNA and telomere dynamics during cell differentiation^77^.

In our analysis, specific HOX targets of DEHMs were selected, and a network of HOX-miRNAs was generated using Cytoscape 3.9.1^78,79^ (https://cytoscape.org/). The interaction between miR-196a/miR-196b with *HOX* genes, namely, *HOXA5, HOXA7, HOXA9, HOXB7,* and *HOXC8* (Supplementary Table S8, Fig. 7c) was observed. Thus, the construction of this regulatory network could offer relevant cues in the oncogenic progression of HNSCC.

### Construction of HOX cluster functional network in HNSCC

The regulatory network of DEHGs, their corresponding 55 target genes (Fig. 7d) and DEHMs were constructed using Cytoscape 3.9.1^78–81^ (Fig. 7e). The complex interactive landscape confirmed that the HOX cluster genes are co-regulatory. The deregulation in upstream HOX factors could eventually impact all the downstream targets, causing deregulation of crucial signaling pathways and driving carcinogenesis.

## Discussion

The aberrant expression of HOX cluster genes bears clinical significance for diagnosis, prognosis, and treatment in HNSCC^16,46^. However, the potential role of *HOX* genes as disease-specific markers of circulating tumor cells is gaining credence^48,82^. The present study investigated the oncogenic HOX genes cluster to elucidate their molecular mechanism and determine their clinical application in HNSCC. In the present study, 16 DEHGs were identified as upregulated in HNSCC (TCGA-HNSCC dataset). Notably, more than two *HOX* genes from each of the four HOX clusters (A, B, C, and D) were deregulated in HNSCC.

In addition, the paralogous *HOX* genes belonging to group 9 (*HOXA9*, *HOXB9*, and *HOXC9*) were found to be aberrantly expressed in HNSCC, similar to that of the HOX13 paralogue as demonstrated earlier in OSCC^24^. Further, the protein level expression of DEHGs in HNSCC has shown concordance with their mRNA level in HNSCC. PPIN showed an interaction among HOXC4, HOXC5, HOXC6, and HOXB7, suggesting that the co-expression of these DEHGs might be critically involved in oncogenic progression^41–44^.

The aberrant expression of *HOX* genes in malignancies has been attributed to various genetic and epigenetic alterations^16^. Interactions between HOX transcription factors and cancer-associated loci due to SNVs/mutations may precede cancer predisposition and progression^16^. The DEHGs exhibited the heterozygous amplification (CNV) in the HNSCC dataset, amongst which *HOXA9, HOXA10,* and *HOXA11* are the most frequent, indicating that the alteration in the gene expression of *HOX* genes might be due to CNVs^83^.

Further, epigenetic modifications can alter the activity of cell signaling pathways and have been associated with the acquisition of drug resistance^84^. Characterization of the epigenome thus helps predict the tumor's biological behavior and the treatment outcomes. The promoter DNA methylation events impact the expression of *HOX* genes to drive tumorigenesis^5,17,85–87^. The in silico analysis of the TCGA–HNSCC dataset showed that out of nine DEHGs that were aberrantly methylated, five of them showed an inverse correlation between DNA methylation and gene expression, implying the promoter DNA methylation-mediated transcriptional regulation (Table 1, Supplementary Fig. S1). Notably, *HOXD10* was found to be hypermethylated in HNSCC^83^. The increase in *HOXD10* expression despite the promoter hypermethylation is rather unusual as promoter hypermethylation is typically associated with reduced expression^83^^,^^88^. While the promoter DNA hypermethylation of *HOXA9* contributes to the HNSCC metastasis^89^. On the contrary, a recent study on OSCC supports our observation that both *HOXB13* and *HOXA9* genes were regulated by gene amplification^83^. However, altered epigenetic regulation of *HOX* genes by histone modification in OSCC cells was positively correlated with the oncogenic transformation of oral keratinocytes^86^.

To determine the impact of the aberrant expression of *HOX* genes on biological processes, we have performed the pathway enrichment analysis. Studies on the utility of *HOX* gene as a biomarker for metastasis and invasion are just emerging^90^. Out of the 16 DEHGs in our computational analysis, all except *HOXD13* showed association with invasion and metastasis. The involvement of *HOXD10, HOXD11, HOXD1, HOXC4, HOXC10,* and *HOXA11,* in the cell cycle and the EMT, confirm their role in the cancer-related signaling pathways. Further, evidence supports our observation that *HOXA9, HOXA10, HOXB7, HOXB9, HOXB13, HOXC8, HOXC9, HOXD1,* and *HOXD10* are involved in angiogenesis^91,92^. Hence, determining the differential expression and functional enrichment of *HOX* genes in different clinical stages and HPV-associated HNSCC is critical. Researchers have reported that higher expression of *HOXA1* in HPV-positive HNSCC leading to a reduction in the CD + T cell infiltration may be an independent prognostic factor in HNSCC^48^. Assessment of the expression of each DEHGs regarding HPV status revealed five DEHGs *HOXB13, HOXC5, HOXC6, HOXC9,* and *HOXD11* which showed a significant difference in expression between HPV-positive and HPV-negative HNSCC tumors, implying their role in cancer-related molecular events in HPV-positive HNSCC.

Likewise, the differential expression of *HOX* gene has been identified in the different clinical stages of breast cancer (BC)^15,62^. Our computational analysis predicts five *HOX* genes, *HOXA10, HOXB9, HOXC4, HOXC8*, and *HOXD1* differentiating specific stages of HNSCC.

Survival analysis based on the expression of DEHGs revealed that overexpression of *HOXB9*, *HOXC5,* and *HOXC6* was associated with a poor prognosis in HNSCC. In particular, *HOXA1* expression was associated with poor pathological grade, advanced stage, and perineural invasion in HNSCC^48^. Furthermore, HOX paralogous genes, *HOXA13* and *HOXD13,* have been aberrantly expressed in OSCC, with *HOXD13* expression inversely relating to overall survival^24^.

After understanding the clinical significance of DEHGs, it is crucial to assess newer algorithms to identify suitable drug candidates to enable genotype-guided prescription for improved drug efficiency. Among the approved drugs that are shown to interact with the DEHGs, some of them have sensitized the cells having higher levels of *HOXD13, HOXD10,* and *HOXB13* expression, while *HOXB7* overexpression offered resistance to several drugs of GDSC^65^. Moreover, the research must be focused on targeting only oncogenic *HOX* genes by carefully considering their property of functional redundancy. Hence, researchers proposed an alternative approach that successfully restrained the tumor growth, enhanced the apoptosis, and reduced the recurrence of prostate cancer (PCa) by developing a peptide, namely HXR9, which functions as a competitive antagonist of HOX-PBX interaction^64,93^.

As transcription factors (TF), HOX proteins regulate many cellular processes by modulating the expression of key downstream target genes^94^, and their identification may aid in developing targeted therapies. Further, it has been elucidated that direct HOX targets participate preferentially in diverse cellular functions such as organogenesis, cell-differentiation, cell cycle progression, cell adhesion, migration, and apoptosis either by trans-activation or by repression^95^. Identifying and characterizing the HOX transcription factors and their downstream targets are paramount in discovering potential cancer biomarkers in HNSCC. In silico detection of the TF binding motifs to explore the downstream-regulated molecules of *HOX* genes and to map it to the driver genes of HNSCC identified 55 possible HOX-target driver genes of HNSCC, which were found to be enriched explicitly in 36 different key signaling pathways such as the MAPK pathway, VEGF pathway, hypoxia response, PI3K pathway, Wnt –signaling pathway, P53 pathway, and apoptosis. Amongst them, *CASP8, TFDP2,* and *IL18* were identified as the downstream targets of at least four DEHGs in HNSCC. *CASP8* is one of the frequently mutated genes in HNSCC. The loss of *CASP8* sensitizes cancer cells to necroptosis via a complex regulatory mechanism^96^. Further, *CASP8* has a dual role as an oncogene and tumor suppressor gene and thus functions as a potential therapeutic target^97^. Functional validation and deciphering the mechanism of action may aid in the discovery of novel clinical biomarkers in HNSCC. Furthermore, overexpression of the inflammatory cytokine IL-18, secreted by macrophages play a crucial role in the inflammatory and immune response and could mediate tumor suppression through the activation of natural killer (NK) and T cells. Overexpression of IL-18 has been shown to induce proliferation, migration, metastasis, immune escape, and angiogenesis^98^. Notably, elevated serum levels of IL-18 in HNSCC play a potential role in an immunological response^99^. Thus, characterization of the HOX/IL-18 interaction and their downstream pathways may offer a new therapeutic strategy in HNSCC. Another HOX target identified in this study is the TFDP family of transcription factors that form complexes with E2F to regulate cell-cycle progression from the G1 to S phase^100^. However, as six out of 16 DEHGs have TFDP2 as a downstream target, it might have biological importance in HNSCC. Taken together, these downstream targets of HOX proteins support the hypothesis that the HOX network regulates multiple cellular pathways involved in carcinogenesis and thus showed their systemic effect on the development of cancer hallmarks.

However, HOX cluster genes not only interact with other protein-coding genes but also with ncRNAs embedded within cluster^19^. The oncogenic and tumor-suppressive HOX cluster-embedded miRNAs have been shown to regulate cell proliferation, metastasis, and recurrence in different cancers, including HNSCC^67,101–105^. The four HOX clusters contain six miRNAs that belong to two kinds of miRNA families, namely miR-196 and miR-10. The genes of miR-196 and miR-10 are transcribed at three (miR‐196b, miR‐196a‐1, and miR‐196a‐2) and two (miR-10a and miR-10b) different locations in four HOX clusters, respectively. In addition to these miRNAs, miR-615 in the HOXC cluster may serve as an excellent biomarker in HNSCC prognosis^106^. Our analysis confirmed the downregulation of miR-10b and upregulation of all other HOX cluster-embedded miRNAs in HNSCC tissue samples.

Interestingly, increased levels of miR-196a and miR-196b have been detected in saliva samples from HNSCC patients. Transfection of cancer-associated fibroblasts (CAFs) with specific pre-miR precursors showed that both miR-196a and miR-196b elicit cell-specific responses in target genes^107^. Further, it has been demonstrated that miR-196 a/b and miR-10b consistently play an oncogenic role in HNSCC by promoting cell proliferation^101^. The coordinated upregulation of *HOXB9* and miR-196a was observed in the HNSCC samples, positively correlating with migration and invasion^108^. Similarly, we found the upregulation of *HOX* genes which act as targets of DEHMs in HNSCC. This might be due to the miRNA-mediated novel mechanism driving the gene activation, in which miRNA interacts with the promoter region to recruit transcription factors and RNA-polymerase-II^108,109^. When we retrieved the targets of DEHMs using different databases, we observed their involvement in cancer-related pathways. Careful evaluation of targets of DEHMs confirmed that co-regulated miRNAs share the common targets. While some of the targets were derived from the HOX cluster, others were the common targets of DEHGs. Constructing a network of DEHGs-DEHMs and target genes that were common to HOX-transcription factors, we could understand the interactive role of all the protein-coding genes and miRNAs organized on all the four HOX clusters in HNSCC. Alongside, the inclusion of lncRNAs embedded in HOX cluster may augur well for future analysis.

Overall, this study provides a comprehensive overview of the *HOX* genes and the embedded miRNA network in the context of HNSCC using genomics, epigenomics, and pharmaco-genomic approaches. Even though genome-wide studies have revealed the functional role of HOX-proteins, to our knowledge, this is the first attempt to construct a HOX cluster network and predict its co-regulatory role in HNSCC. Hence, functional categorization of tumors and identification of molecular targets regulated by the HOX cluster may be of translational relevance. Experimental validation of their molecular mechanism may be helpful in clinical evaluation and enable the discovery of an improved therapeutic regimen in HNSCC.

## Methods

### Data retrieval and identification of differentially expressed HOX genes

DEHGs from the TCGA-HNSCC (sample size: normal n = 44, tumor n= 520) data set were identified using a freely available online tool, the TACCO database. TACCO provides information about DEGs in 26 different cancer types from the TCGA datasets. TACCO can be used for pathway analysis and prognostic model construction using input gene lists^25,110^. We identified the DEHGs between tumor and normal tissue samples in the TCGA-HNSCC data set, using the "select DEGs" option with a parameter cutoff *p*-value ≤ 0.05 and expression of log2 fold change >  + 2 and <  − 2, calculated using EBSeq, Wilcoxon rank-sum test and multiple test correction^25^. The expression of individual DEHGs was further verified using the UALCAN tool, which determines relative gene expression across normal and tumor samples in TCGA datasets of 31 cancer types by performing a Sample *t*-test (*p*-value ≤ 0.05)^28^.

### Identification of genetic variations in DEHGs

Genetic variations such as SNVs, missense mutations, nonsense mutations, in-frame mutations, and CNVs in the TCGA dataset were analyzed using the GSCALite database. The onco-plot depicting the percentage of SNVs in DEHGs in the TCGA-HNSCC dataset was obtained^26^. The database allows the analysis of the desired gene list for their differential expression, methylation, genomic variations, pathway, and drug sensitivity in 33 different cancers of TCGA datasets^26^. These observations were further verified using the TCGA-firehose legacy HNSC dataset (n = 530), accessed using the cBioPortal^27,111^. It is an open-access tool for visualizing multidimensional cancer genomics data in 20 different cancer types^27^.

### Identification of methylation-regulated DEHGs

Identification of methylation-regulated DEHGs in the TCGA-HNSCC dataset (sample size: normal n = 50, tumor n = 528) was carried out using the UALCAN online web server^28^. Beta values of 0 and 1 were considered unmethylated and completely methylated, respectively, and values between 0.7–0.5 and 0.3–0.25 with a *p*-value of ≤ 0.05 were significantly hypermethylated and hypomethylated, respectively. Further, we used the DNMIVD to verify the same, which considers |beta difference|> 0.2 as differential methylation between normal (n = 50) and tumor samples (n = 530) by performing an unpaired *t*-test and adjusting the *p*-value by Benjamini/Hochberg method^29^. The common genes were further checked for their correlation between gene expression and methylation by performing Pearson's correlation analysis with cutoff Pearson |r value|> 0.3 and Bonferroni *p*-value ≤ 0.05 using DNMIVD.

### Cross-validation of DEHGs in independent datasets and the expression of DEHGs at the protein level

The gene expression of DEHGs was further tested in independent datasets using the Oncomine Research Edition online tool^30^. The gene expression was verified by setting the fold change threshold |1.5| with the *p*-value ≤ 0.05 computed using the Student’s *t*-test in HNSCC datasets. Further, we analyzed the expression of 16 DEHGs at the protein level using the HPA database^39^.

### Construction of PPIN

PPIN of the DEHGs was constructed using the STRING version 11.5, with the highest confidence score of > 0.7^40^. The web server provides the protein–protein interaction data by considering the gene co-occurrence, gene co-expression, and protein homology, which were experimentally determined.

### Pathway analysis of DEHGs

Pathway enrichment analysis of DEHGs was downloaded as a heatmap percentage from GSCALite, using the TCGA-HNSC dataset^26^. Individual genes were assessed for their specific role in cancer using CHAT^51^. This tool can retrieve and organize the cancer-associated cellular processes from PubMed and determine the strength of association between cancer hallmarks and the query gene. Statistics were computed using the Fisher’s exact test or Chi-squared test followed by Bonferroni correction.

### Targets of the DEHGs, HNSCC-driver genes, pathway analysis, and association with cancer hallmarks

The driver genes of HNSCC were queried using the DriverDBv3 online tool^52^. As HOX proteins function as transcription factors, they would be expected to regulate the transcription of multiple target genes precisely. TF2DNA database^53^ was explored to identify the downstream targets of DEHGs (connectivity *p*-value: 0.0001). Gathered from experimental and theoretical sources, this database provides information about transcription factors and their downstream targets for five model organisms, including humans^53^. Predicted target genes of each HOX transcription factor were compared with driver genes of HNSCC using Venny 2.1.0 https://bioinfogp.cnb.csic.es/tools/venny/. The overlapping target genes driving HNSCC were subjected to pathway analysis using PANTHER web server^54,55^. The identified targets were tested for association with cancer hallmarks using the CHG database^56^. By determining the degree, betweenness, and clustering coefficient of the nodes, the database annotates the potential roles of hallmark genes in 34 different cancer phenotypes^56^.

### Association of DEHGs in HPV infection, staging, risk prediction, and prognosis

The association between DEHGs with HPV and tumor stage was evaluated using the UALCAN database, which estimates the difference in the transcript per million (TPM) values by performing a Sample *t*-test^28^. The *HOX* genes were analyzed for their differential expression in HPV-positive and HPV-negative tumors, and between the various cancer stages with a *p*-value of ≤ 0.05 were considered statistically significant.

To analyze the prognostic significance of DEHGs, a random forest algorithm was employed using the TACCO online tool^25^. The prognostic prediction model stratifies the patients into a high and low-risk group based on the log-rank test. The database considers the patients with a prediction score larger than 0.5 as high risks, while those lower than 0.5 are considered low risk^25^. The prediction model of survival data was generated by creating a KM plot and the patient survival data at each time point for every gene. The prognostic value of individual genes was tested using the GEPIA2, which calculates the strength of correlation between the expression of individual genes via OS and DFS analysis in TCGA data sets^63^. The association between the variables and survival rate was determined by calculating the Hazard ratio based on Cox Proportional-Hazards regression with a 95% confidence interval.

### Interaction between DEHGs and drug sensitivity

The association between gene expression and drug sensitivity was measured using the GSCALite portal, which retrieves drug-gene interaction data from the GDSC database^26,65,66^. GDSC determines the correlation between the expression profile of genes in cancer cell lines and their drug sensitivity (IC50) for small molecule drugs by determining the area under the dose–response curve (AUC) for the drugs^112^. Hence, positive correlation means cell lines with higher gene expression levels are resistant to the therapeutic drugs.

### Identification of DEHMs in HNSCC

The individual gene expression of the six HOX cluster-embedded miRNAs in the TCGA-HNSCC dataset (sample size: normal n = 44; primary tumor n = 482) was retrieved from the UALCAN database^28^ and further verified using starBase v2.0^113^ (sample size: normal n = 44; primary tumor n = 497). The differential miRNA expression between normal and tumor samples with a *p*-value ≤ 0.05 was considered statistically significant.

### Target genes of DEHMs and their functional enrichment analysis (FEA)

The target genes of DEHMs were retrieved from mirDB, http://mirdb.org/^114^ and mirDIP 4.1, https://ophid.utoronto.ca/mirDIP/databases^115^. The common miRNA targets were analyzed if experimentally validated using the miRTarbase database, http://mirtarbase.cuhk.edu.cn/^116^. The *HOX* genes which themselves act as target genes of corresponding DEHMs were identified. Following their expression analysis in the HNSCC dataset using UALCAN, a regulatory network of HOX-miRNA was constructed using Cytoscape^81^. Further, the miRNA-miRNA co-expression network was constructed using the CoMeTa tool^71^. Pathway enrichment analysis of the upregulated and downregulated miRNA target genes was performed in the Enrichr tool^72^, using the KEGG web server^73^, which computes the statistical significance (*p*-value ≤ 0.05) by performing Fisher’s exact test.

### Construction of functional regulatory network

To determine the entire molecular landscape of HOX cluster, the complex regulatory network of DEHGs, DEHMs, and their target genes in HNSCC (*p*-value ≤ 0.05) was constructed using Cytoscape^81^.

## Supplementary Information

Supplementary Information.

## Data Availability

All data retrieved and analyzed in this study are included in the manuscript, and additional information has been provided as supplementary files.

## Abbreviations

AUC: Area under the dose–response curve
BC: Breast cancer
CAFs: Cancer-associated fibroblasts
CASP8: Caspase-8
CDKN2A: Cyclin-Dependent Kinase Inhibitor 2A
CESC: Cervical squamous cell carcinoma
CHAT: Cancer Hallmarks Analytics Tool
CHG: Cancer Hallmark Genes
CNVs: Copy Number Variations
DEGs: Differentially Expressed Genes
DEHGs: Differentially Expressed HOX genes
DEHMs: Differentially Expressed HOX cluster-embedded miRNAs
DFS: Disease-free survival
DNMIVD: DNA methylation interactive visualization database
EMT: Epithelial-mesenchymal transition
GDSC: Genomics of Drug Sensitivity in Cancer
GEPIA: Gene Expression Profiling Interactive Analysis
GSCALite: Gene Set Cancer Analysis
HGNC: HUGO gene Nomenclature Committee
HNSCC: Head and Neck Squamous Cell Carcinoma
HPA: Human Protein Atlas
HPV: Human papillomavirus
IHC: Immunohistochemistry
IL-18: Interleukin-18
KM-plot: Kaplan–Meier survival plot
miRNA: MicroRNA
ncRNAs: Noncoding RNAs
OS: Overall survival
PANTHER: Protein Analysis Through Evolutionary Relationships
PI3KCA: Phosphatidylinositol-4,5-Bisphosphate 3-Kinase Catalytic Subunit Alpha
PPIN: Protein–protein interaction network
PCa: Prostate Cancer
RNA activation: RNAa
SNVs: Single nucleotide variations
STRING: Search Tool for the Retrieval of Interacting Genes
TACCO: Transcriptome Alterations in Cancer Omnibus
TCGA: The Cancer Genome Atlas
TFDP2: Transcription Factor Dimerization Partner 2
TPM: Transcript Per Million
TP53: Tumor suppressor gene 53
UTR: Untranslated region
