# Supplementary material for: Integrated computational analysis reveals HOX genes cluster as oncogenic drivers in head and neck squamous cell carcinoma
Source: Sci Rep. 2022 May 13;12:7952. doi: 10.1038/s41598-022-11590-1 (PMC9106698; doi:10.1038/s41598-022-11590-1)
Supplement: Supplementary file 1 — Supplementary Information. [file 41598_2022_11590_MOESM1_ESM.pdf]

Integrated computational analysis reveals HOX genes cluster as oncogenic drivers in head and neck squamous cell carcinoma

Running Head: HOX gene in HNSCC

U Sangeetha Shenoy M.Sc.[a], Richard Morgan Ph. D.[b], Keith Hunter Ph. D.[c], Shama Prasada Kabekkodu Ph. D.[a], Raghu Radhakrishnan Ph. D.[d*]

[a]Department of Cell and Molecular Biology, Manipal School of Life Sciences, Manipal Academy of Higher Education, Manipal, Karnataka, 576104, India
[b]School of Biomedical Sciences, University of West London, London, W5 5RF, UK

[c]Academic Unit of Oral and Maxillofacial Medicine and Pathology, School of Clinical Dentistry, University of Sheffield, Sheffield, S10 2TA, UK
[d]Department of Oral Pathology, Manipal College of Dental Sciences, Manipal Academy of Higher Education, Manipal – 576104, India

Funding

This work was supported by the DBT/Wellcome Trust India Alliance Fellowship, (Grant number - IA/CPHI/18/1/503927) awarded to Raghu Radhakrishnan.

*Corresponding Author

Raghu Radhakrishnan MDS, Ph.D.

Wellcome Trust DBT IA Fellow and Professor

Department of Oral Pathology

Manipal College of Dental Sciences

Manipal Academy of Higher Education, Manipal – 576104, India

Email: - raghu.ar@manipal.edu

ORCID: 0000-0003-0088-4777

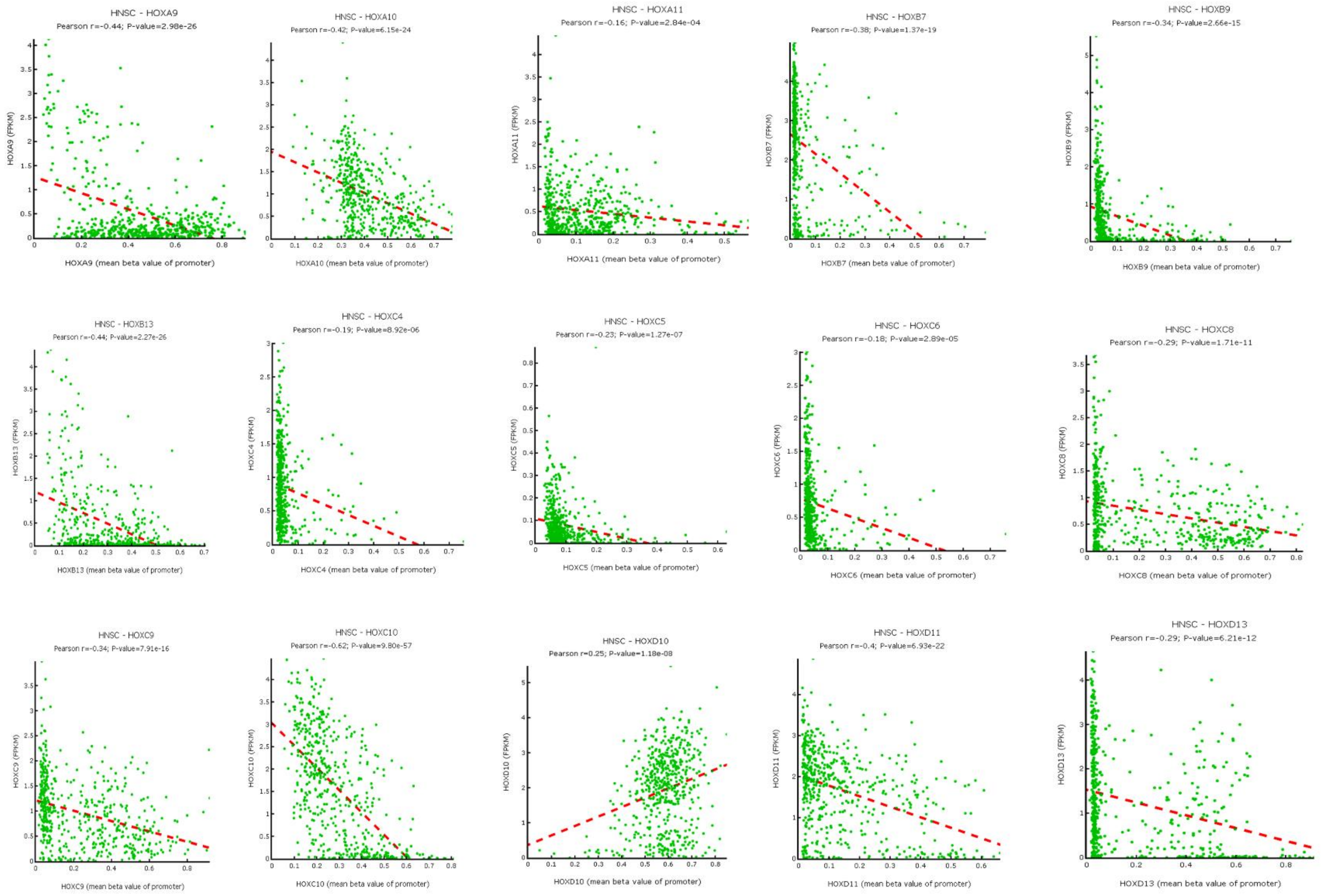

Supplementary Fig. S1: Determination of relation between gene expression and promoter DNA methylation using Pearson's correlation analysis with cutoff |Pearson r| value > 0.3 and p value: ≤0.05 considered statistically significant.

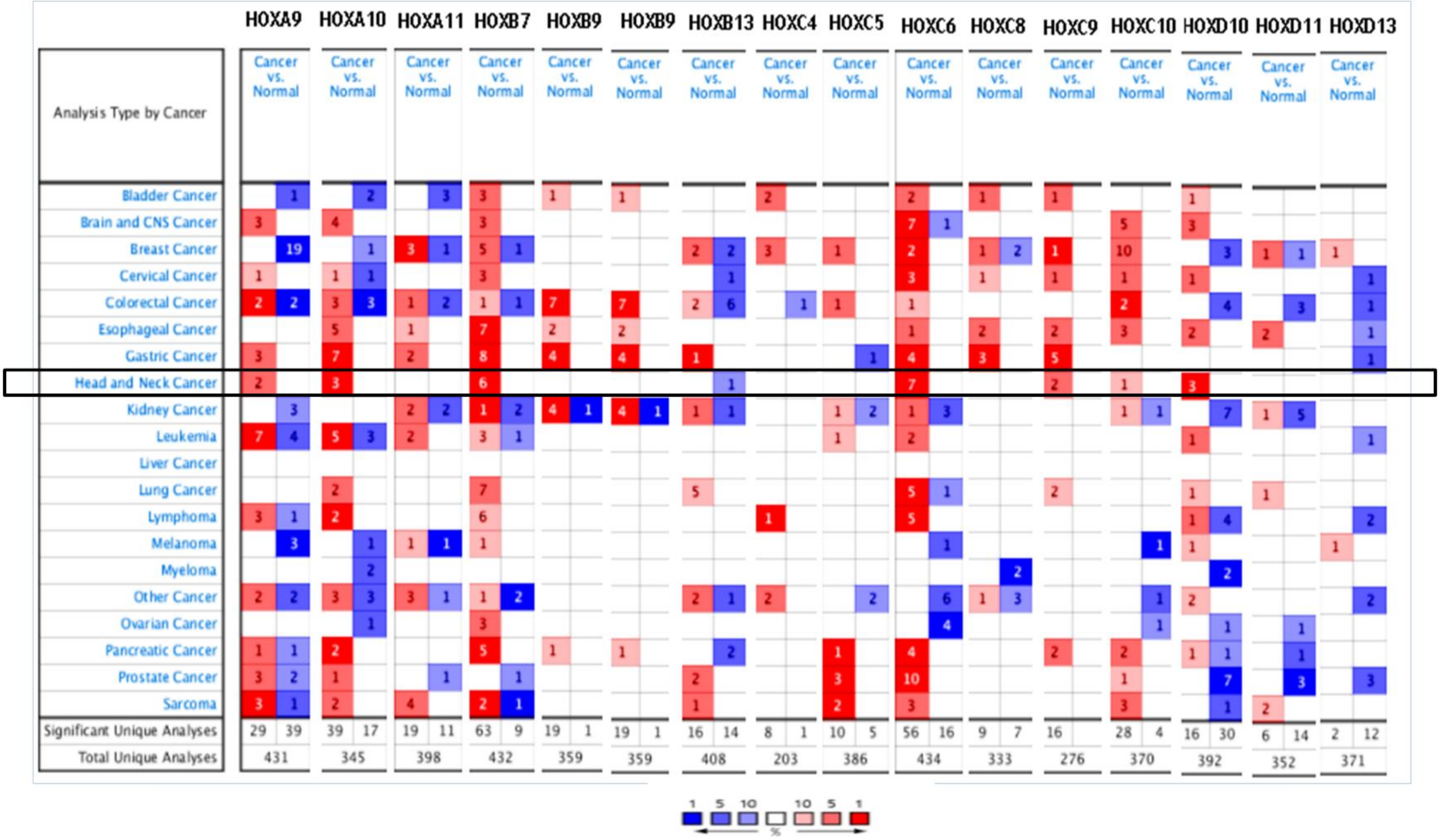

Supplementary Fig. S2: The plots represent the mRNA expression of HOX genes in HNSCC cancer tissue against normal tissue (Highlighted in the black box), according to the Oncomine database in several independent datasets. The red color indicates overexpression and the blue color indicates underexpression. Color intensity indicates the best rank of the gene in the analysis. The number in each box represents the number of analyses that meet our threshold.

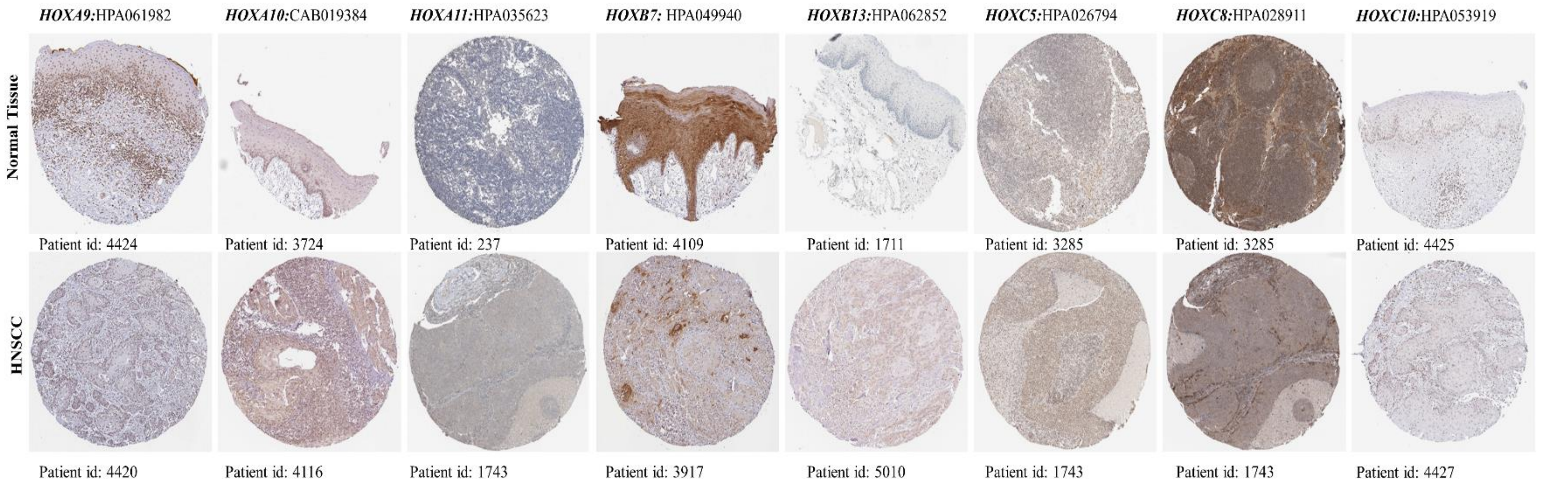

Supplementary Fig. S3: The IHC images represent the expression of HOX–proteins in HNSCC.

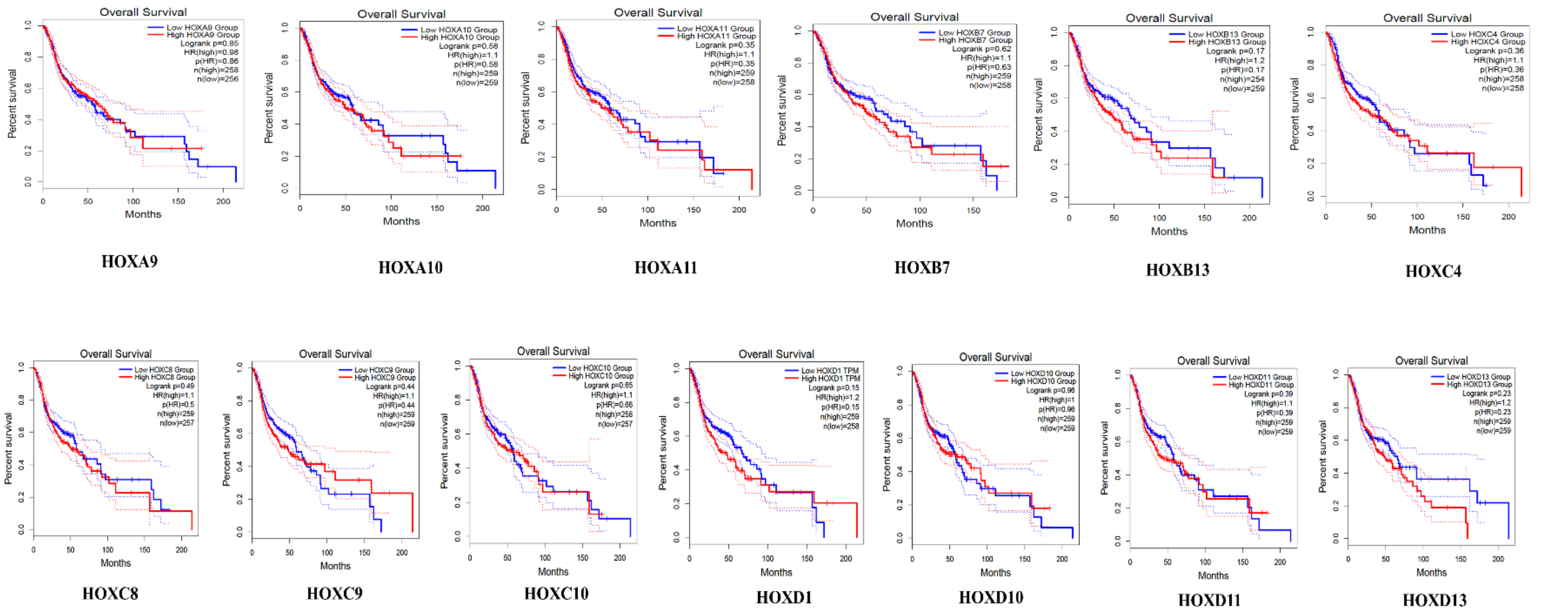

Supplementary Fig. S4: Overall survival analysis of DEHGs in HNSCC (p value: ≤0.05).

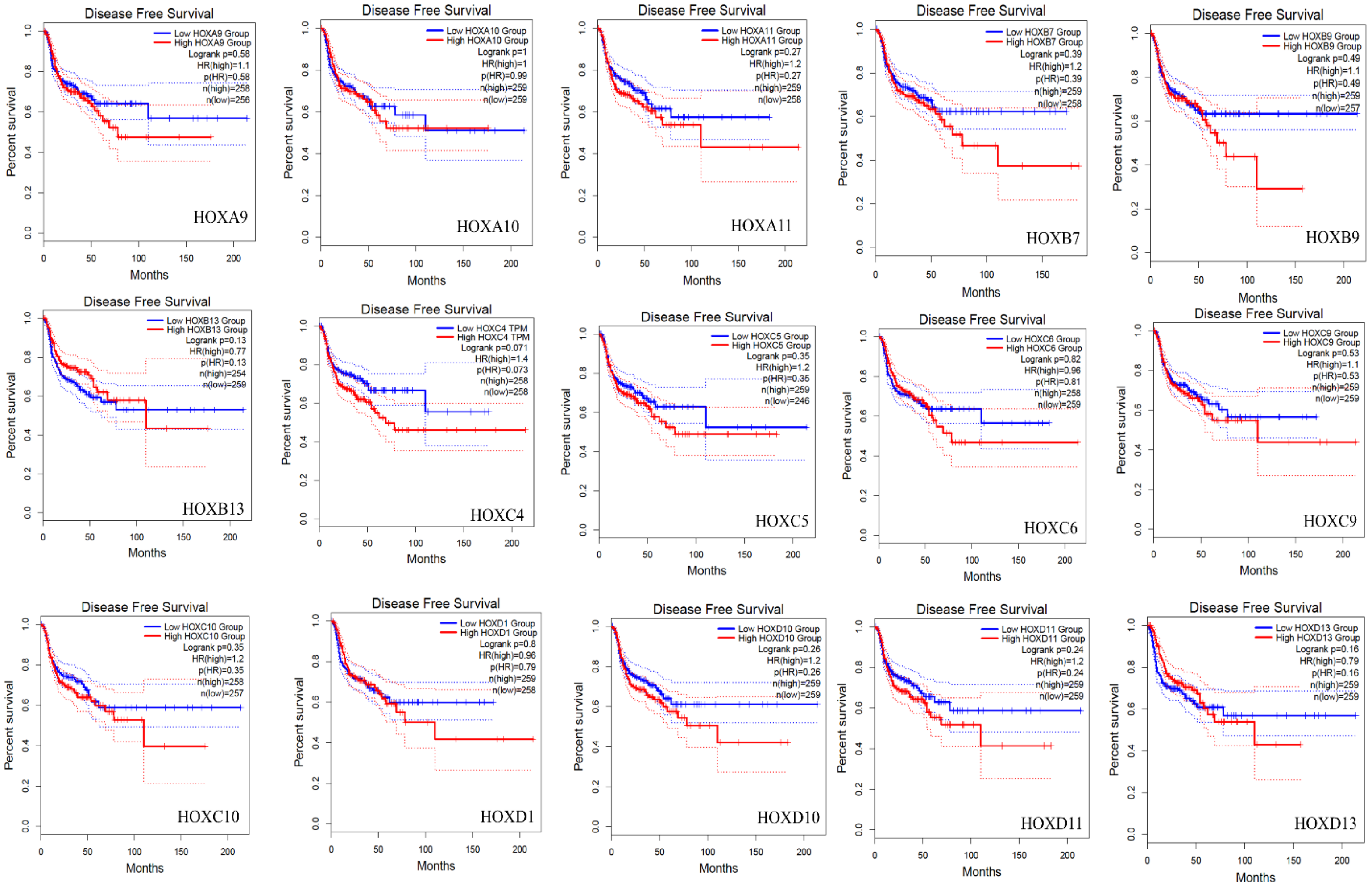

Supplementary Fig. S5: Disease-free survival analysis of DEHGs in HNSCC (p value: ≤0.05).

Supplementary Table S1: DEHGs in HNSCC retrieved from TACCO database

HOX genes	Fold change	log2(Fold change)	Mean TPM* (tumor)	Mean TPM* (normal)	p-value	Adjusted p-value
HOXA9	4.3	2.11	61.1	14.19	3.84E-07	0.00000142
HOXA10	11.76	3.56	178.77	15.2	5.88E-23	1.89E-20
HOXA11	28.91	4.85	35.46	1.23	1.51E-21	1.89E-19
HOXB7	11.13	3.48	217.01	19.5	2.90E-16	6.62E-15
HOXB9	71.6	6.16	65.77	0.92	5.05E-19	2.29E-17
HOXB13	21.55	4.43	55.65	2.58	0.0000064	0.0000192
HOXC4	5.41	2.43	48.94	9.05	3.11E-17	8.79E-16
HOXC5	9.79	3.29	16.38	1.67	2.47E-20	1.87E-18
HOXC6	13.75	3.78	59.47	4.33	1.21E-23	6.67E-21
HOXC8	16.85	4.07	42.15	2.5	4.48E-22	7.29E-20
HOXC9	13.03	3.7	44.62	3.42	2.11E-22	4.18E-20
HOXC10	17.02	4.09	150.6	8.85	1.00E-16	2.49E-15
HOXD1	4.19	2.07	28.4	6.77	7.06E-11	5.20E-10
HOXD10	11.39	3.51	196.59	17.25	2.92E-20	2.11E-18
HOXD11	14.27	3.83	203.3	14.25	7.17E-22	1.07E-19
HOXD13	21.93	4.45	115.65	5.27	7.61E-17	1.94E-15

Note: The HOX genes which showed significant difference in the gene expression between normal (n=44) and tumor (n=520) samples retrieved from TACCO database with cutoff p-value ≤ 0.05 and expression of log 2-fold change >+2 and <-2, calculated using EBSeq, Wilcoxon rank-sum test and multiple test correction.

Supplementary Table S2: Genetic variations associated with DEHGs in TCGA-HNSCC firehose legacy dataset (n=530) verified using CBioPortal

**HOX genes**	**Sample ID**	**Mutation Type**	**Protein Change**	**Variant Type**
HOXA11	TCGA-CV-7099-01	Missense_Mutation	R293K	SNP
	TCGA-CV-7263-01	Missense_Mutation	Q95H	SNP
HOXB7	TCGA-CV-5440-01	Missense_Mutation	E155G	SNP
	TCGA-CV-6961-01	Nonsense_Mutation	E155*	SNP
HOXB13	TCGA-CV-7568-01	Missense_Mutation	D244N	SNP
	TCGA-KU-A66S-01	Missense_Mutation	Y130S	SNP
HOXC4	TCGA-CN-4723-01	Missense_Mutation	E188K	SNP
	TCGA-F7-A624-01	Missense_Mutation	A250V	SNP
	TCGA-F7-A61V-01	Missense_Mutation	M3K	SNP
	TCGA-BA-A6DI-01	Missense_Mutation	M209K	SNP
HOXC6	TCGA-CQ-6227-01	Missense_Mutation	R110M	SNP
	TCGA-CV-5978-01	Missense_Mutation	S131L	SNP
	TCGA-TN-A7HL-01	Missense_Mutation	E107K	SNP
	TCGA-CQ-7071-01	Missense_Mutation	S85L	SNP
HOXC8	TCGA-BB-8596-01	Missense_Mutation	S133L	SNP
	TCGA-CV-A461-01	Missense_Mutation	S80I	SNP
HOXC9	TCGA-CN-4735-01	Missense_Mutation	E208K	SNP
HOXC10	TCGA-QK-A6VB-01	Missense_Mutation	R183H	SNP
HOXD10	TCGA-CR-6477-01	Missense_Mutation	Y151C	SNP
	TCGA-BB-4223-01	Missense_Mutation	R326P	SNP
	TCGA-CV-7427-01	Missense_Mutation	Q156H	SNP
	TCGA-F7-7848-01	Missense_Mutation	R89G	SNP
	TCGA-CQ-7064-01	Missense_Mutation	D83H	SNP
	TCGA-CR-7383-01	Missense_Mutation	R153T	SNP
HOXD13	TCGA-BA-6873-01	Missense_Mutation	V183L	SNP
	TCGA-CV-7424-01	Missense_Mutation	G195D	SNP
	TCGA-QK-A6VB-01	In_Frame_Del	A71del	DEL

Supplementary Table S3: Differentially methylated *HOX* genes in HNSCC

	According to DNMIVD database			According to UALCAN database		
DEHGs	Beta value difference	p-value	Differential methylation type	Beta value difference	p-value	Differential methylation type
HOXA9	0.26069	4.20E-18	Hypermethylation	0.261	<1E-12	Differential methylation
HOXA10	0.102516	4.93E-08	Differential methylation	0.077	1.62E-12	Differential methylation
HOXA11	0.0652961	1.55E-05	Differential methylation	0.17	1.62E-12	Differential methylation
HOXB7	0.0336509	0.022	Differential methylation	0.04	<1E-12	Differential methylation
HOXB9	0.00867853	0.594	Not Significant	0.064	<1E-12	Differential methylation
HOXB13	0.0832255	6.95E-05	Differential methylation	0.087	<1E-12	Differential methylation
HOXC4	-0.0100261	0.317	Not Significant	0.167	1.62E-12	Differential methylation
HOXC5	-0.0115	0.274	Not Significant	0.116	1.62E-12	Differential methylation
HOXC6	-0.0100261	0.317	Not Significant	0.151	<1E-12	Differential methylation
HOXC8	0.201527	2.75E-09	Hypermethylation	0.142	1.62E-12	Differential methylation
HOXC9	0.120107	1.18E-04	Differential methylation	0.098	1.62E-12	Differential methylation
HOXC10	-0.0320692	0.136	Not Significant	0.018	3.34E-07	Differential methylation
HOXD1	0.037674	0.103	Not Significant	-0.012	0.000341	Differential methylation
HOXD10	0.298189	8.29E-86	Hypermethylation	0.364	<1E-12	Hypermethylation
HOXD11	0.0555936	0.013	Differential methylation	-0.006	0.0783	Not Significant
HOXD13	0.154665	3.39E-06	Differential methylation	0.227	1.62E-12	Differential methylation

Note: By carefully comparing the differential methylation pattern in HNSCC using DNMIVD (sample size: normal n= 50, tumor n-=528) and UALCAN (sample size: normal n= 50, tumor n-=528) databases, the common differentially methylated HOX genes were considered for further analysis.

Supplementary Table S4: DEHGs and their target genes driving HNSCC

**Transcription factors**	**Targets driving HNSCC**
HOXA9	*CASP8, MPZL3, IL18, CARD17, ABCG4, TFDP2, CCR2, EOMES, LPP, ZNF701, ZNF83*
HOXA11	*CASP8, MAPK24, TFDP2, PC, ZNF422*
HOXB9	*ELAVL2, PANK2, SNX18, TXNRD1*
HOXB13	*CREBBP, DNAH5, LEKR1, PTPRM*
HOXC4	*AKT1, ANK2, BRAF, CASP8, MAP2K4, LETM2, CASP4, FEZ1, TFDP2, LPP, TXNRD1, ZNF486, ZNF83, ZNF93*
HOXC8	*CASP8, MAP2K4, ANAPC15, SHANK2, MPZL3, IL18, SERPINB13, TFDP2, LEKR1, PLQ, PC, NRN1*
HOXC9	*KRAS, PIK3R1, SIRPB1, ELAVL2, IL18, CD226, TFDP2, PLS1, DDX3Y, C11orf53, CNP, PANK2, PCDHB4, TXNRD1, ZNF93*
HOXD10	*CASP8, FBXW7, FAM86C1, NAALADL2, SIRPB1, MPZL3, CASP4, IL18, CARD17, TFDP2, CCR2, EOMES, LPP, SNX18, UBR7, ZNF83*
HOXD11	*CASP8, IFNE, IQCB1, KCNMB2, PCOLCE2, APPL2, TBX18*
HOXD13	*ARHGEF26*

Supplementary Table S5: Clinical significance of HOX genes in HPV-associated HNSCC

DEHGs	Normal (Median TPM)	HPV +ve (Median TPM)	HPV-ve (Median TPM)	p-value
HOXA9	0.026	0.424	0.225	0.35952
HOXA10	0.122	5.604	2.886	0.20138
HOXA11	0	0.21	0.315	0.42808
HOXB7	0.22	11.568	8.898	0.159938
HOXB9	0	0.522	0.335	0.125078
HOXB13	0	1.045	0.027	0.00057539
HOXC4	0.11	1.469	1.052	0.42162
HOXC5	0.015	0.671	0.358	0.022162
HOXC6	0.03	3.579	0.971	0.000195727
HOXC8	0	0.637	0.487	0.144703
HOXC9	0.027	3.083	1.2	0.0052845
HOXC10	0.012	3.141	2.505	0.085198
HOXD1	0.116	0.553	0.441	0.146876
HOXD10	0.171	1.783	7.032	0.70004
HOXD11	0.048	6.081	18.606	0.047423
HOXD13	0	2.572	1.021	0.125201

Note: The HOX genes which showed differential expression pattern between normal (n=44), HPV-positive (HPV+ve ; n=41)) and HPV-negative (HPV-ve; n=80) tissue samples, with p value ≤0.05 were considered as statistically significant.

Supplementary Table S6: Clinical significance of HOX genes in stage stratification of HNSCC

DEHGs	Normal (Median TPM)	Stage 2 (Median TPM)	Stage 3 (Median TPM)	Stage 4 (Median TPM)	Stage 5 (Median TPM)	Cancer stages	p-value
HOXA10	0.146	2.596	3.336	3.732	4.212	stage 1 -vs-stage 4	3.32E-02
HOXB9	0	0.179	0.334	0.195	0.564	stage 1 -vs-stage 4	7.54E-03
HOXC8	0	0.733	0.638	0.726	0.608	stage 1 -vs-stage 4	3.92E-03
HOXC4	0.122	1.407	1.36	1.509	1.511	stage 2 -vs-stage 4	2.56E-02
HOXD1	0.119	0.348	0.527	0.439	0.52	stage 1 -vs-stage 2	2.34E-03
						stage 1 -vs-stage 3	7.43E-04
						stage 1 -vs-stage 4	8.34E-07

Note: The HOX genes which showed significant difference (p≤0.05) in the gene expression between different clinical stages of HNSCC (Sample size: normal: n=44; Stage 1: n=27; Stage 2: n=71; Stage 3: n=81; Stage 4: n-=264) have been considered.

Supplementary Table S7: DEHMs and their targets in HNSCC

DEHMs in HNSCC	Upregulated target genes	Downregulated target genes
**miR-196b /hsa-miR-196b-5p**	*SRRT,SMCR8,RDX, RCC2,NR6A1, NAP1L1,LRIG3,IGF2BP3, IGF2BP1,IGDCC4,HOXC8 ,HOXB7, HOXA9,HOXA5,HMGA2, HMGA1,GGA3, FAS, FAM104A,EXOC8, DFFA, CALM, BRAP,BACH1,ACER2,KCTD21*	*YOD1, TGFBR3, PBX1, SUOX, RGL2, CDKN1B*
**miR-196a-1/hsa-miR-196a-5p**	*ACER2,ARHGAP28,BACH1,BRAP,CALM1,CCDC47,CTPS1,DFFA,EXOC8,FAM104A,GATA6,GGA3,HMGA1,HMGA2,HOXA5,HOXA7,HOXA9,HOXB7,IGDCC4,IGF2BP1,IGF2BP3,KCTD21,LRIG3,MAP3K21,MIEF1,NAP1L1,NEMP1,NR6A1,NXPE3,PHC3,RAB29,RCC2,RDH11,RDX,RTL8A,SMARCAD1,SMC3,SMCR8,SRRT,TRPC3ZNF850*	*NTN4,TSPAN12,RGL2 ,TGFBR3,CDKN1B,BCL11A,YOD1,CPEB3, GLTP,SUOX*
**miR-615/ hsa-miR-615-3p**	*TOMM7,NUP62,LRPAP1,CBX6,SUPT16H*	
**miR-196a-2/hsa-miR-196a-3p**	*NUFIP2,SEC31A,EIF4G2,AMMECR1L,DFFB,IRGQ,TRPV1*	*PCBD2*
**miR-10b/hsa-miR-10b-5p**	*TFAP2C,CNOT6,CRLF3,CREB1,CRK,ARSK,MAPRE1,LIX1L,NR2C2,BCL2L11,SDC1,HOXD10,ACTG1,SON,SNX4,NCOR2,TRA2B,PIK3CA,CSGALNACT1,ZNF445,PAFAH1B1,ANXA7,RNF2,NCOA6,TPM4,LSS,URGCP,MSL3,XPNPEP3*	*RORA,TIAM1,TRIM2, CSMD1,NR4A3,KLF4, ID4,ACVR2A*

Supplementary Table S8: DEHMs targeting the HOX cluster genes HNSCC

**HOX cluster-embedded miRNAs**	**Location**	**Expression in HNSCC**	**Upregulated target genes in HOX cluster**
miR-196b /hsa-miR-196b-5p	Between *HOXA9* and *HOXA10*	↑	*HOXC8*, *HOXB7*, *HOXA9 and HOXA5*
miR-10a/hsa-miR-10a-5p	Upstream from *HOXB4*	Non-significant	*HOXA1*, *HOXA2*
miR-196a-1/hsa-miR-196a-5p	Near *HOXB9*	↑	*HOXA5*, *HOXA7*, *HOXA9*, *HOXB7* and *HOXC8*
miR-615/ hsa-miR-615-3p	HOXC Cluster	↑	-
miR-196a-2/hsa-miR-196a-3p	Between *HOXC9* and *HOXC10*	↑	-
miR-10b/hsa-miR-10b-5p	Upstream from *HOXD4*	↓	*HOXD10*

Note: ↑-upregulation; ↓-downregulation
